# Nuclear global spaces of ultradifferentiable functions in the matrix weighted setting

**DOI:** 10.1007/s43037-020-00090-x

**Published:** 2020-10-19

**Authors:** Chiara Boiti, David Jornet, Alessandro Oliaro, Gerhard Schindl

**Affiliations:** 1 Dipartimento di Matematica e Informatica, Università degli Studi di Ferrara, Via Machiavelli n. 30, 44121 Ferrara, Italy; 2 Instituto Universitario de Matemática Pura y Aplicada IUMPA, Universitat Politècnica de València, Camino de Vera, s/n, 46071 Valencia, Spain; 3 Dipartimento di Matematica, Università degli Studi di Torino, Via Carlo Alberto n. 10, 10123 Torino, Italy; 4 Fakultät für Mathematik, Universität Wien, Oskar-Morgenstern-Platz n. 1, 1090 Wien, Austria

**Keywords:** Weight matrices, Ultradifferentiable functions, Sequence spaces, Nuclear spaces, 46A04, 46A45, 26E10

## Abstract

We prove that the Hermite functions are an absolute Schauder basis for many global weighted spaces of ultradifferentiable functions in the matrix weighted setting and we determine also the corresponding coefficient spaces, thus extending the previous work by Langenbruch. As a consequence, we give very general conditions for these spaces to be nuclear. In particular, we obtain the corresponding results for spaces defined by weight functions.

## Introduction

The systematic study of nuclear locally convex spaces began in 1951 with the fundamental dissertation of Grothendieck [20] to classify those infinite dimensional locally convex spaces which are not normed, suitable for mathematical analysis. Among the properties of a nuclear space, the existence of a Schwartz kernel for a continuous linear operator on the space is of crucial importance for the theory of linear partial differential operators. In our setting of ultradifferentiable functions, this fact helps, for instance, to study the behaviour (propagation of singularities or wave front sets) of a differential or pseudodifferential operator when acting on a distribution. See, for example, [1, 7, 16, 17, 33, 38] and the references therein.

Since the middle of the last century, several authors have studied the topological structure of global spaces of ultradifferentiable functions and, in particular, when the spaces are nuclear. See [31], or the book [19]. More recently, the first three authors in [9] used the isomorphism established by Langenbruch [28] between global spaces of ultradifferentiable functions in the sense of Gel’fand and Shilov [18] and some sequence spaces to see that under the condition that appears in [11, Corollary 16(3)] on the weight function $\omega$ (as in [12]) the space $\mathcal {S}_{(\omega )}({\mathbb {R}}^d)$ of rapidly decreasing ultradifferentiable functions of Beurling type in the sense of Björck [3] is nuclear. However, there was the restriction that the powers of the logarithm were not allowed as admissible weight functions. Later, the authors of the present work proved in [10] that $\mathcal {S}_{(\omega )}({\mathbb {R}}^d)$ is nuclear for any weight function satisfying $\log (t)=O(\omega (t))$ and $\omega (t)=o(t)$ as *t* tends to infinity. The techniques used in [10] come especially from the field of time–frequency analysis and a mixture of ideas from [7, 21, 22, 38]. In both [9] and [10], we use (different) isomorphisms between that space $\mathcal {S}_{(\omega )}({\mathbb {R}}^d)$ and some sequence space and prove that $\mathcal {S}_{(\omega )}({\mathbb {R}}^d)$ is nuclear by an application of the Grothendieck–Pietsch criterion [32, Theorem 28.15]. Very recently, Debrouwere, Neyt and Vindas [14, 15] (cf. [27] for related results about local spaces), using different techniques have extended our previous results in a very general framework. In [14], they characterize when mixed spaces of Björck [3] of Beurling type or of Roumieu type are nuclear under very mild conditions on the weight functions. In [15], using weight matrices in the sense of [37], the same authors characterize the nuclearity of generalized Gel’fand–Shilov classes which extend their previous work [14] and treat also many other mixed classes defined by sequences.

The aim of the present paper is twofold. On the one hand, we extend the work of Langenbruch [28] to the matrix weighted setting in the sense of [37, 40]. In particular, we prove that the Hermite functions are a Schauder basis of many global weighted spaces of ultradifferentiable functions. Moreover, we determine the coefficient spaces corresponding to this Hermite expansion (Theorem 1). These results are applied to spaces defined by weight functions $\mathcal {S}_{[\omega ]}({\mathbb {R}}^d)$, being $[\omega ]=(\omega )$ (Beurling setting) or $[\omega ]=\{\omega \}$ (Roumieu setting). Hence, we extend part of the previous work of Aubry [2] to the several variables case. As a consequence we are able to generalize our previous study [9, 10] about the nuclearity of the space $\mathcal {S}_{(\omega )}({\mathbb {R}}^d)$ to global spaces of ultradifferentiable functions defined by weight matrices (Corollary 2). An application to particular matrices gives that $\mathcal {S}_{(\omega )}({\mathbb {R}}^d)$ is nuclear when $\omega (t)=o(t^2)$ as *t* tends to infinity. Similarly, we also prove the analogous result for the Roumieu setting, namely that $\mathcal {S}_{\{\omega \}}({\mathbb {R}}^d)$ is nuclear when $\omega (t)=O(t^2)$ as *t* tends to infinity (see Theorem 6 for both results). For weights of the form $\omega (t)=\log ^\beta (1+t)$ with $\beta >1$, our results hold and, hence, we generalize the results of [28] to spaces that could not be treated there since, as is easily deduced from [11, Example 20], $\mathcal {S}_{[(M_p)_p]}({\mathbb {R}})\ne \mathcal {S}_{[\omega ]}({\mathbb {R}})$ for any sequence of positive numbers $(M_p)_{p\in {\mathbb {N}}}$ in the sense of [26] (see Remark 4). We do not treat here the classical case $\omega (t)=\log (1+t)$, for which $\mathcal {S}_{(\omega )}({\mathbb {R}})=\mathcal {S}({\mathbb {R}})$, the Schwartz class, because in this case infinitely many entries of our weight matrices are not well defined. However, the results presented here are already well known for the Schwartz class.

The classes of functions treated in [15] are in general different from ours. In fact, here we consider spaces of functions *f* that are bounded in the following sense: for some (or any) $h>0$, there is $C>0$ such that for all $x\in {\mathbb {R}}^d$ and every multi-indices $\alpha$ and $\beta$, we have

$$ |x^\alpha \partial ^\beta f(x)|\le C h^{|\alpha +\beta |}M_{\alpha +\beta }\qquad \qquad (A). $$

And we pass to the matrix setting for the multi-sequence $(M_\alpha )_\alpha$, i.e. we make $M_\alpha ^\lambda$ depend also on a parameter $\lambda >0$ (see the precise definition in the next section). In [15], the authors consider spaces of functions *f* bounded in the following sense: there is $C>0$ such that for all $x\in {\mathbb {R}}^d$ and every multi-index $\beta$ they have

$$ |w(x) \partial ^\beta f(x)|\le C M_\beta\qquad \qquad (B), $$

where *w* is a positive continuous function. They pass to the matrix setting by making $M_\beta ^\lambda$ and $w^\lambda$ depend on the same parameter $\lambda >0$. Hence, taking unions (Roumieu setting) or intersections (Beurling setting) in $\lambda$ in the situation (*A*) gives different classes of functions than in the situation (*B*) in general. On the other hand, it is a very difficult problem to determine when the classes treated in this work are non-trivial, a question not considered in [14, 15]. We characterize in a very general way (Propositions 2 and 3) when the Hermite functions are contained in our classes and this fact is closely related to classes being non-trivial. Indeed, we can deduce from our results that, in the Beurling setting, the space $\mathcal {S}_{(\omega )}({\mathbb {R}}^d)$ contains the Hermite functions if and only if $\omega (t)=o(t^2)$ as *t* tends to infinity (Corollary 3). However, it is not difficult to see from the uncertainty principle [23, Theorem] that $\mathcal {S}_{(\omega )}({\mathbb {R}}^d)=\{0\}$ when $t^2=O(\omega (t))$ as *t* tends to infinity. In the same way, in the Roumieu case, the space $\mathcal {S}_{\{\omega \}}({\mathbb {R}}^d)$ contains the Hermite functions if and only if $\omega (t)=O(t^2)$ as *t* tends to infinity (Corollary 3), but again from [23, Theorem] we can deduce $\mathcal {S}_{\{\omega \}}({\mathbb {R}}^d)=\{0\}$ when $t^2=o(\omega (t))$ as *t* tends to infinity. For more information on the uncertainty principle for $\mathcal {S}_{[\omega ]}({\mathbb {R}}^d)$ where, as stated above, $[\omega ]=(\omega )$ or $\{\omega \}$, see the nice introduction to the paper of Aubry [2] and the references therein. Moreover, our classes are well adapted for Fourier transform (Corollary 1). We should also mention that throughout this paper we assume, on the multi-sequence $(M_\alpha )_\alpha$, that $(M_{\alpha })^{1/|\alpha |}$ tends to infinity when $|\alpha |$ tends to infinity, which is stronger than the condition $\inf _{\alpha \in {\mathbb {N}}^d_0}(M_\alpha /M_0)^{1/|\alpha |}>0$ considered in [26, Def. 3.1] (for the one-dimensional case). The reason is that it is not clear how the results read when the associated function is infinite (see Remark 1).

The paper is organized as follows: in the next section, we give some necessary definitions, in Sect. 3 we introduce the classes under study in the matrix weighted setting and establish the analogous conditions to [28] to determine in Sect. 4 when the Hermite functions belong to our classes. In Sect. 5, we introduce the suitable matrix sequence spaces and prove that they are isomorphic to our classes, which is the fundamental tool to see that our spaces are nuclear. We finally apply these results to the particular case of spaces defined by weight functions in Sect. 6.

## Preliminaries

In what follows, for given $t=(t_1,\dots ,t_d)\in {\mathbb {R}}^d$, we are setting $|t|_{\infty }:=\max _{1\le j\le d}|t_j|$. We briefly recall from [26] those basic notions about sequences $\mathbf {M}=(M_p)_{p\in {\mathbb {N}}_0}$, for ${\mathbb {N}}_0:={\mathbb {N}}\cup \{0\}$, that we need in what follows. A sequence $(M_p)_p$ is called *normalized* if $M_0=1$. For a normalized sequence $\mathbf {M}=(M_p)_p$, the *associated function* is denoted by

2.1 $$ \omega _{\mathbf {M}}(t)=\sup _{p\in {\mathbb {N}}_0}\log \frac{|t|^p}{M_p},\qquad t\in {\mathbb {R}}. $$

We say that $(M_p)_p$ satisfies the *logarithmic convexity* condition (*M*1) of [26] if

2.2 $$M_p^2\le M_{p-1}M_{p+1},\qquad p\in {\mathbb {N}}. $$

The following lemma is well known (see Lemmas 2.0.6 and 2.0.4 of [39] for a proof).

### Lemma 1

*Let* $(M_p)_{p\in {\mathbb {N}}_0}$ *be a normalized sequence satisfying* (2.2). *Then*

- $$M_jM_k\le M_{j+k}$$ for all $$j,k\in {\mathbb {N}}_0$$;
- $$p\mapsto (M_p)^{1/p}$$ is increasing;
- $$\liminf _{p\rightarrow +\infty }(M_p)^{1/p}>0$$.

From Lemma 1(c) and [26, Prop. 3.2], we have that a normalized sequence $\mathbf {M}=(M_p)_p$ satisfies (2.2) if and only if

2.3 $$M_p=\sup _{t>0}\frac{t^p}{\exp \omega _{\mathbf {M}}(t)},\qquad p\in {\mathbb {N}}_0. $$

We say that $(M_p)_p$ satisfies the *stability under differential operators* condition $(M2)'$ of [26] if

2.4 $$ \exists A,H\ge 1\ \forall p\in {\mathbb {N}}_0:\quad M_{p+1}\le AH^pM_p, $$

and $(M_p)_p$ satisfies the stronger *moderate growth* condition (*M*2) of [26] if

2.5 $$ \exists A\ge 1\ \forall p,q\in {\mathbb {N}}_0:\quad M_{p+q}\le A^{p+q}M_pM_q. $$

The following lemma extends [26, Proposition 3.4] for two sequences. We give the proof for the convenience of the reader.

### Lemma 2

*Let* $\mathbf {M}=(M_p)_{p\in {\mathbb {N}}_0}$ and $\mathbf {N}=(N_p)_{p\in {\mathbb {N}}_0}$ *be two normalized sequences satisfying* (2.2). *Then the following conditions are equivalent:*

(i) $$\exists A\ge 1\ \forall p\in {\mathbb {N}}_0: \quad M_{p+1}\le A^{p+1}N_p$$.
(ii) $$\exists A\ge 1, B>0\ \forall t>0: \quad \omega _{\mathbf {N}}(t)+\log t\le \omega _{\mathbf {M}}(At)+B$$.

### Proof

If (i) is satisfied, then, for all $t>0$,

$$ te^{\omega _\mathbf {N}(t)}=t\sup _{p\in {\mathbb {N}}_0}\frac{t^p}{N_p}\le \sup _{p\in {\mathbb {N}}_0}\frac{(At)^{p+1}}{M_{p+1}} \le \sup _{p\in {\mathbb {N}}_0}\frac{(At)^p}{M_p}=e^{\omega _\mathbf {M}(At)}. $$

Conversely, if (*ii*) holds, then, by (2.3),

$$\begin{aligned} N_p=&\sup _{t>0}\frac{t^p}{\exp \omega _\mathbf {N}(t)}\ge \sup _{t>0} \frac{t^{p+1}}{e^B\exp \omega _\mathbf {M}(At)}\\ =&e^{-B}\sup _{s>0}\frac{(s/A)^{p+1}}{\exp \omega _\mathbf {M}(s)} =\frac{e^{-B}}{A^{p+1}}M_{p+1}.\end{aligned}$$

$$\square$$

Now, we consider sequences $\mathbf {M}=(M_\alpha )_{\alpha \in {\mathbb {N}}_0^d}$ of positive real numbers for multi-indices $\alpha \in {\mathbb {N}}_0^d$. As in the one-dimensional case, we say that $(M_\alpha )_{\alpha \in {\mathbb {N}}_0^d}$ is normalized if $M_0=1$. We recall condition (3.7) of [28]

2.6 $$\exists A\ge 1\ \forall \alpha ,\beta \in {\mathbb {N}}_0^d: \quad M_\alpha M_\beta \le A^{|\alpha +\beta |}M_{\alpha +\beta }. $$

Condition (2.4) takes in this setting the form (see [28, (2.1)])

2.7 $$\begin{aligned} \exists A\ge 1\ \forall \alpha \in {\mathbb {N}}_0^d, 1\le j\le d:\quad M_{\alpha +e_j}\le A^{|\alpha |+1}M_{\alpha }, \end{aligned}$$

and (2.5) turns into

2.8 $$\begin{aligned} \exists A\ge 1\ \forall \alpha ,\beta \in {\mathbb {N}}_0^d: \quad M_{\alpha +\beta }\le A^{|\alpha +\beta |}M_{\alpha }M_{\beta }. \end{aligned}$$

Now, for $t\in {\mathbb {R}}^d$, we denote

2.9 $$\begin{aligned} {\mathbb {N}}_{0,t}^d:=\{\alpha \in {\mathbb {N}}^d_0 : \alpha _j=0\ \text {if}\ t_j=0,\ j=1,\dots ,d\}. \end{aligned}$$

The *associated weight function* of a normalized $\mathbf {M}=(M_\alpha )_{\alpha \in {\mathbb {N}}_0^d}$ is given by

$$\begin{aligned} \omega _\mathbf {M}(t)=\sup _{\alpha \in {\mathbb {N}}_{0,t}^d}\log \frac{|t^\alpha |}{M_\alpha },\;\;\;t\in {\mathbb {R}}^d, \end{aligned}$$

where by convention $0^0:=1$. Note that for a normalized sequence we have $\omega _{\mathbf {M}}(0)=0$.

### Remark 1

As it has already been pointed out in the geometric construction in [30, Chap. I] for the one dimensional weight function (see (2.1)), we have that $\omega _\mathbf {M}(t)<+\infty$ for all $t\in {\mathbb {R}}^d$ if and only if $\lim _{|\alpha |\rightarrow \infty }(M_{\alpha })^{1/|\alpha |}=+\infty$.

First, assume that $\omega _\mathbf {M}(t)<+\infty$ for all $t\in {\mathbb {R}}^d$. Hence for all $t=(t_1,\dots ,t_d)\in {\mathbb {R}}^d$ satisfying $t_{\min }:=\min _{1\le j\le d}|t_j|\ge 1$ we have that there exists some *C* (depending only on *t*) such that $\log \frac{|t^\alpha |}{M_\alpha }\le C$ for all $\alpha \in {\mathbb {N}}_0^d$. So $t_{\min }^{|\alpha |}\le |t_1^{\alpha _1}\cdots t_d^{\alpha _d}|=|t^{\alpha }|\le e^CM_{\alpha }$ for all $\alpha \in {\mathbb {N}}_0^d$ and now let $t_{\min }\rightarrow +\infty$.

Conversely, let $\lim _{|\alpha |\rightarrow \infty }(M_{\alpha })^{1/|\alpha |}=\infty$ and so for any $A>0$ large, we can find some $C>0$ large enough such that $A^{|\alpha |}\le CM_{\alpha }$. Since $|t^{\alpha }|\le |t|^{|\alpha |}$ for all $t\in {\mathbb {R}}^d$ and $\alpha \in {\mathbb {N}}_0^d$, we see that for any given $t\in {\mathbb {R}}^d$ we get $\frac{|t^\alpha |}{M_\alpha }\le \frac{|t|^{|\alpha |}}{M_{\alpha }}\le C$ for some $C>0$ and all $\alpha \in {\mathbb {N}}_0^d$.

### Lemma 3

*Let* $\mathbf {M}=(M_\alpha )_{\alpha \in {\mathbb {N}}_0^d}$. *Then, for all* $h>0$ *and* $\alpha \in {\mathbb {N}}_0^d$,

2.10 $$\begin{aligned} M_\alpha h^{|\alpha |}\ge \sup _{t\in {\mathbb {R}}^d}|t^\alpha | e^{-\omega _\mathbf {M}(t/h)}. \end{aligned}$$

### Proof

Fix $\alpha \in {\mathbb {N}}^d_0$ and $h>0$; we write ${\mathbb{R}}^{d}_{\alpha} := \{ t \in {\mathbb{R}}^{d}  : t_j \ne 0 {\text{ for }} \alpha_j \ne 0, j=1,\dots, d \}$. Then for $t\in {\mathbb {R}}^d\setminus {\mathbb {R}}^d_\alpha$, we have $t^\alpha =0$, and so it is enough to prove that

2.11 $$M_\alpha h^\alpha \ge \sup_{t\in {\mathbb{R}}^d_\alpha} |t^\alpha| e^{-\omega_{\mathbf{M}}(t/h)}.$$

We have

$$\begin{aligned} \frac{1}{\displaystyle \sup _{t\in {\mathbb {R}}^d_\alpha }|t^\alpha | e^{-\omega _\mathbf {M}(t/h)}}=&\inf _{t\in {\mathbb {R}}^d_\alpha } \frac{e^{\omega _\mathbf {M}(t/h)}}{|t^\alpha |} =\inf _{t\in {\mathbb {R}}^d_\alpha }\frac{\exp {\displaystyle \sup _{\beta \in {\mathbb {N}}_{0,t}^d} \log \frac{\big |\left( \frac{t}{h}\right) ^\beta \big |}{M_\beta }}}{|t^\alpha |}\\ =&\inf _{t\in {\mathbb {R}}^d_\alpha } \frac{1}{|t^\alpha |}\sup _{\beta \in {\mathbb {N}}_{0,t}^d}\frac{\big |\left( \frac{t}{h}\right) ^\beta \big |}{ M_\beta }; \end{aligned}$$

observe that $\alpha \in {\mathbb {N}}^d_{0,t}$ and so, choosing $\beta =\alpha$, we get

$$\begin{aligned} \frac{1}{\displaystyle \sup _{t\in {\mathbb {R}}^d_\alpha }|t^\alpha | e^{-\omega _\mathbf {M}(t/h)}}\ge \inf _{t\in {\mathbb {R}}^d_\alpha }\frac{|t^\alpha |}{|t^\alpha | h^{|\alpha |}M_\alpha } =\frac{1}{h^{|\alpha |}M_\alpha }, \end{aligned}$$

which proves (2.11), and then the proof is complete.

Note that if $\omega _\mathbf {M}(t/h)=+\infty$, then (2.10) is clear and so we could restrict in the estimates above to all $t\in {\mathbb {R}}^d_\alpha$ such that $\omega _\mathbf {M}(t/h)$ is finite.$\square$

In the following, we use two normalized sequences as above $\mathbf {M}=(M_\alpha )_{\alpha \in {\mathbb {N}}_0^d}$ and $\mathbf {N}=(N_\alpha )_{\alpha \in {\mathbb {N}}_0^d}$ and we compare them in the sense:

$$\begin{aligned} \mathbf {M}\le \mathbf {N}\quad \text{ if }\quad M_\alpha \le N_\alpha , \quad \alpha \in {\mathbb {N}}_0^d. \end{aligned}$$

This clearly implies

$$\begin{aligned} \omega _\mathbf {N}(t)\le \omega _\mathbf {M}(t),\quad t\in {\mathbb {R}}^d. \end{aligned}$$

In [28], Langenbruch uses his condition (1.2) to prove that the Hermite functions belong to the spaces considered there. In the present paper we need, for the same reason, a mixed condition that involves two sequences:

2.12 $$\begin{aligned} \exists H,C,B>0\ \forall \alpha ,\beta \in {\mathbb {N}}_0^d:\quad \alpha ^{\alpha /2}M_\beta \le BC^{|\alpha |}H^{|\alpha +\beta |}N_{\alpha +\beta }. \end{aligned}$$

### Remark 2

Condition (2.12) yields that $\lim _{|\alpha |\rightarrow \infty }(N_{\alpha })^{1/|\alpha |}=+\infty$. Indeed, since by convention $0^0=1$ and by definition $\alpha ^{\alpha /2}:=\alpha _1^{\alpha _1/2}\cdots \alpha _d^{\alpha _d/2}$, from (2.12) with $\beta =0$ we get (recall $|\alpha |_\infty :=\max _{1\le j\le d}\alpha _j$) that

$$\begin{aligned} N_\alpha ^{1/|\alpha |}\ge&B^{-\frac{1}{|\alpha |}}C^{-1}H^{-1}(\alpha ^{\alpha /2})^{1/|\alpha |} =B^{-\frac{1}{|\alpha |}}C^{-1}H^{-1} (\alpha _1^{\alpha _1/2}\cdots \alpha _d^{\alpha _d/2})^{1/|\alpha |}\\ \ge&B^{-\frac{1}{|\alpha |}}C^{-1}H^{-1}(|\alpha |_\infty ^{|\alpha |_\infty /2})^{1/|\alpha |} \ge B^{-\frac{1}{|\alpha |}}C^{-1}H^{-1}\left( \frac{|\alpha |}{d}\right) ^{\frac{1}{2d}}\rightarrow +\infty . \end{aligned}$$

## Global ultradifferentiable functions in the matrix weighted setting

In this section, we consider matrices of normalized sequences $(M^{(\lambda )}_\alpha )_{\lambda >0,\alpha \in {\mathbb {N}}_0^d}$ of real positive numbers:

3.1 $$\begin{aligned} \begin{aligned} \qquad \mathcal {M}:=\{(\mathbf {M}^{(\lambda )})_{\lambda >0}:\mathbf {M}^{(\lambda )}= (M^{(\lambda )}_\alpha )_{\alpha \in {\mathbb {N}}_0^d},\ M^{(\lambda )}_0=1, \\\mathbf {M}^{(\lambda )}\le \mathbf {M}^{(\kappa )}\,\text{ for } \text{ all }\,0<\lambda \le \kappa \}. \end{aligned} \end{aligned}$$

We call $\mathcal {M}$ a *weight matrix* and consider matrix weighted global ultradifferentiable functions of Roumieu type defined as follows (from now on $\Vert \cdot \Vert _\infty$ denotes the supremum norm): first, for a given normalized sequence $\mathbf {M}$, we set

$$\begin{aligned} \mathcal {S}_{\{\mathbf {M}\}}:=&\Big \{f\in C^\infty ({\mathbb {R}}^d):\ \exists C,h>0, \ \ \Vert f\Vert _{\infty ,\mathbf {M},h}:=\sup _{\alpha ,\beta \in {\mathbb {N}}_0^d} \frac{\Vert x^\alpha \partial ^\beta f\Vert _\infty }{h^{|\alpha +\beta |} M_{\alpha +\beta }}\le C\Big \},\\ \mathcal {S}_{(\mathbf {M})}:=&\{f\in C^\infty ({\mathbb {R}}^d):\ \forall h>0\ \exists C_h>0, \ \ \Vert f\Vert _{\infty ,\mathbf {M},h}\le C_h\}, \end{aligned}$$

endowed with the inductive limit topology in the Roumieu setting (which may be thought countable if we take $h\in {\mathbb {N}}$) and with the projective limit topology in the Beurling setting (countable for $h^{-1}\in {\mathbb {N}}$). Next, we define the matrix type spaces as follows:

$$\begin{aligned} \mathcal {S}_{\{\mathcal {M}\}}:=&\bigcup _{\lambda>0}\mathcal {S}_{\{\mathbf {M}^{(\lambda )}\}} =\{f\in C^\infty ({\mathbb {R}}^d):\ \exists C,h,\lambda>0,\ \ \Vert f\Vert _{\infty ,\mathbf {M}^{(\lambda )},h}\le C\},\\ \mathcal {S}_{(\mathcal {M})}:=&\bigcap _{\lambda>0}\mathcal {S}_{(\mathbf {M}^{(\lambda )})}\\ =&\{f\in C^\infty ({\mathbb {R}}^d):\ \forall h,\lambda>0\ \exists C_{\lambda ,h}>0,\ \ \Vert f\Vert _{\infty ,\mathbf {M}^{(\lambda )},h}\le C_{\lambda ,h}\}, \end{aligned}$$

again endowed with the inductive limit topology in the Roumieu setting (which may be thought countable if we take $\lambda ,h\in {\mathbb {N}}$) and endowed with the projective limit topology in the Beurling setting (countable for $\lambda ^{-1},h^{-1}\in {\mathbb {N}}$).

Now we consider different conditions on the weight matrices that we use following the lines of [28]. The next basic condition extends (1.2) of [28] in the Roumieu case and is needed to show that the Hermite functions belong to $\mathcal {S}_{\{\mathcal {M}\}}$ (see Proposition 3):

3.2 $$\begin{aligned} \begin{aligned}&\forall \lambda>0\ \exists \;\kappa \ge \lambda , B,C,H>0\ \forall \alpha ,\beta \in {\mathbb {N}}_0^d: \\&\alpha ^{\alpha /2}M^{(\lambda )}_\beta \le BC^{|\alpha |}H^{|\alpha +\beta |} M^{(\kappa )}_{\alpha +\beta }. \end{aligned} \end{aligned}$$

The analogous condition to (3.2) in the Beurling case, which is needed to show that the Hermite functions belong to $\mathcal {S}_{(\mathcal {M})}$ is the following (see Proposition 3):

3.3 $$\begin{aligned} \begin{aligned}&\forall \;\lambda>0\ \exists \;0<\kappa \le \lambda , H>0\ \forall C>0\ \exists B>0\ \forall \alpha ,\beta \in {\mathbb {N}}_0^d:\\&\alpha ^{\alpha /2}M^{(\kappa )}_\beta \le BC^{|\alpha |}H^{|\alpha +\beta |} M^{(\lambda )}_{\alpha +\beta }. \end{aligned} \end{aligned}$$

### Remark 3

Similarly, as commented in Remark 2 for (2.12), property (3.2) (property (3.3)) yields that $\lim _{|\alpha |\rightarrow \infty }(M^{(\kappa )}_{\alpha })^{1/|\alpha |}=+\infty$ for some $\kappa >0$, and hence for all $\kappa '\ge \kappa$ ($\lim _{|\alpha |\rightarrow \infty }(M^{(\lambda )}_{\alpha })^{1/|\alpha |}=+\infty$ for all $\lambda >0$).

We also need to extend condition (3.7) of [28] to the matrix weighted setting. First, we state it in the Roumieu case:

3.4 $$\begin{aligned} \forall \;\lambda >0\ \exists \;\kappa \ge \lambda , A\ge 1\ \forall \alpha ,\beta \in {\mathbb {N}}_0^d:\ \ M^{(\lambda )}_\alpha M^{(\lambda )}_\beta \le A^{|\alpha +\beta |}M^{(\kappa )}_{\alpha +\beta }; \end{aligned}$$

and in the Beurling case:

3.5 $$\begin{aligned} \forall \;\lambda >0\ \exists \;0<\kappa \le \lambda , A\ge 1\ \forall \alpha ,\beta \in {\mathbb {N}}_0^d:\ \ M^{(\kappa )}_\alpha M^{(\kappa )}_\beta \le A^{|\alpha +\beta |}M^{(\lambda )}_{\alpha +\beta }. \end{aligned}$$

The extensions of condition (2.7) (*mixed derivation closedness properties*) for a weight matrix $\mathcal {M}$ in the Roumieu and Beurling cases read as follows:

3.6 $$\begin{aligned}&\forall \lambda >0\exists \kappa \ge \lambda ,A\ge 1\forall \alpha \in {\mathbb {N}}_0^d,1\le j\le d:\ M^{(\lambda )}_{\alpha +e_j}\le A^{|\alpha |+1}M^{(\kappa )}_\alpha , \end{aligned}$$

3.7 $$\begin{aligned}&\forall \lambda >0\exists 0<\kappa \le \lambda ,A\ge 1\forall \alpha \in {\mathbb {N}}_0^d,1\le j\le d:\ M^{(\kappa )}_{\alpha +e_j}\le A^{|\alpha |+1}M^{(\lambda )}_\alpha . \end{aligned}$$

The following conditions generalize (2.8) to the weight matrix setting:

3.8 $$\begin{aligned}&\forall \lambda >0\exists \kappa \ge \lambda ,A\ge 1\forall \alpha ,\beta \in {\mathbb {N}}_0^d:\ M^{(\lambda )}_{\alpha +\beta }\le A^{|\alpha +\beta |} M^{(\kappa )}_\alpha M^{(\kappa )}_\beta , \end{aligned}$$

3.9 $$\begin{aligned}&\forall \lambda >0\exists 0<\kappa \le \lambda ,A\ge 1\forall \alpha ,\beta \in {\mathbb {N}}_0^d:\ M^{(\kappa )}_{\alpha +\beta }\le A^{|\alpha +\beta |}M^{(\lambda )}_\alpha M^{(\lambda )}_\beta . \end{aligned}$$

It is immediate that for any given matrix $\mathcal {M}$ satisfying (3.8) and (3.4) we can replace in the definition of $\mathcal {S}_{\{\mathcal {M}\}}$ the seminorm $\Vert \cdot \Vert _{\infty ,\mathbf {M}^{(\lambda )},h}$ by

$$\begin{aligned} \sup _{\alpha ,\beta \in {\mathbb {N}}_0^d} \frac{\Vert x^\alpha \partial ^\beta f\Vert _\infty }{h^{|\alpha +\beta |}M^{(\lambda )}_{\alpha }M^{(\lambda )}_{\beta }}. \end{aligned}$$

We have an analogous statement for the class $\mathcal {S}_{(\mathcal {M})}$ under (3.9) and (3.5). When we define the spaces $\mathcal {S}_{\{\mathcal {M}\}}$ or $\mathcal {S}_{(\mathcal {M})}$ with the weighted $L^2$ norms treated below in (3.17), the similar property holds.

### Lemma 4

*Let* $\mathcal {M}$ *be a weight matrix as defined in* (3.1).

*If* (3.6) *holds, then*

3.10 $$\begin{aligned} \begin{aligned}&\forall \;\lambda >0\ \exists \;\kappa \ge \lambda ,B_1,B_2\ge 1\ \forall t\in {\mathbb {R}}^d:\\&(1+|t|)^{2(d+1)}\exp \omega _{\mathbf {M}^{(\kappa )}}(t)\le B_1 \exp \omega _{\mathbf {M}^{(\lambda )}}(B_2t). \end{aligned} \end{aligned}$$

*If* (3.7) *holds, then*

3.11 $$\begin{aligned} \begin{aligned}&\forall \;\lambda >0\ \exists \;0<\kappa \le \lambda ,B_1,B_2\ge 1\ \forall t\in {\mathbb {R}}^d:\\&(1+|t|)^{2(d+1)}\exp \omega _{\mathbf {M}^{(\lambda )}}(t)\le B_1 \exp \omega _{\mathbf {M}^{(\kappa )}}(B_2t). \end{aligned} \end{aligned}$$

### Proof

First, we consider the Roumieu case. By $2(d+1)$ iterated applications of (3.6), we find $\kappa _{2d+2}\ge \kappa _{2d+1}\ge \ldots \ge \kappa _1\ge \lambda >0$ and $A_1,\ldots ,A_{2d+2}\ge 1$ such that, for all $\alpha \in {\mathbb {N}}_0^d$ and $1\le j\le d$,

3.12 $$\begin{aligned} M^{(\lambda )}_{\alpha +2(d+1)e_j}\le&A_1^{|\alpha |+2d+2} M^{(\kappa _1)}_{\alpha +(2d+1)e_j}\nonumber \\ \le&A_1^{|\alpha |+2d+2}A_2^{|\alpha |+2d+1} M^{(\kappa _2)}_{\alpha +2de_j}\nonumber \\ \le&\cdots \le A_1^{|\alpha |+2d+2}A_2^{|\alpha |+2d+1}\cdots A_{2d+2}^{|\alpha |+1} M^{(\kappa _{2d+2})}_{\alpha }\nonumber \\ \le&A^{|\alpha |+2d+2}M^{(\kappa )}_{\alpha } \end{aligned}$$

for $$A:=(\max \{A_1,\ldots ,A_{2d+2}\})^{2d+2}$$ and $$\kappa :=\kappa _{2d+2}$$.

Now, we have for $|t|_\infty \ge 1$,

$$\begin{aligned} (1+|t|)^{2(d+1)}=&\sum _{j=0}^{2(d+1)}\left( {\begin{array}{c}2d+2\\ j\end{array}}\right) |t|^j \le \sum _{j=0}^{2(d+1)}\left( {\begin{array}{c}2d+2\\ j\end{array}}\right) (\sqrt{d}|t|_{\infty })^j\\ \le&d^{d+1}|t|^{2(d+1)}_\infty \sum _{j=0}^{2(d+1)}\left( {\begin{array}{c}2d+2\\ j\end{array}}\right) =(4d)^{d+1}|t|^{2(d+1)}_\infty \end{aligned}$$

since $|t|=\sqrt{t_1^2+\ldots +t_d^2}\le \sqrt{d}|t|_\infty$. Therefore, by the definition of the associated weight function, choosing $\kappa \ge \lambda >0$ as in (3.12), we have, assuming $|t|_\infty =t_j$ for some $1\le j\le d$ and $|t|_\infty \ge 1$:

$$\begin{aligned} (1+|t|)^{2(d+1)}\exp \omega _{\mathbf {M}^{(\kappa )}}(t)\le&(4d)^{d+1}|t_j|^{2(d+1)}\sup _{\alpha \in {\mathbb {N}}_0^d} \frac{|t^\alpha |}{M^{(\kappa )}_\alpha }\\ \le&(4d)^{d+1}\sup _{\alpha \in {\mathbb {N}}_0^d} \frac{|(At)^{\alpha +2(d+1)e_j}|}{M^{(\lambda )}_{\alpha +2(d+1)e_j}}\\ \le&(4d)^{d+1}\sup _{\beta \in {\mathbb {N}}_0^d} \frac{|(At)^{\beta }|}{M^{(\lambda )}_{\beta }} =\,(4d)^{d+1}\exp \omega _{\mathbf {M}^{(\lambda )}}(At). \end{aligned}$$

On the other hand, if $t\in {\mathbb {R}}^d$ with $|t|_\infty \le 1$, then $|t|\le \sqrt{d}$ and hence, for $\kappa$ as in (3.12),

$$\begin{aligned} (1+|t|)^{2(d+1)}\exp \omega _{\mathbf {M}^{(\kappa )}}(t)\le C_\lambda \le C_\lambda \exp \omega _{\mathbf {M}^{(\lambda )}}(At), \end{aligned}$$

with $C_\lambda$ depending on $\lambda$ since $\kappa$ depends on $\lambda$.

We have thus proved (3.10) with $B_1=\max \{(4d)^{d+1},C_\lambda \}$ and $B_2=A$.

In the Beurling case, by $2(d+1)$ iterated applications of (3.7), we find $0<\kappa _{2d+2}\le \kappa _{2d+1}\le \ldots \le \kappa _1\le \lambda$ and $A_1,\ldots ,A_{2d+2}\ge 1$ such that

3.13 $$\begin{aligned} M^{(\lambda )}_\alpha \ge&A_1^{-|\alpha |-1}M^{(\kappa _1)}_{\alpha +e_j} \ge A_1^{-|\alpha |-1}A_2^{-|\alpha |-2}M^{(\kappa _2)}_{\alpha +2e_j}\nonumber \\ \ldots \ge&A_1^{-|\alpha |-1}A_2^{-|\alpha |-2}\cdots A_{2d+2}^{-|\alpha |-2d-2} M^{(\kappa _{2d+2})}_{\alpha +2(d+1)e_j} \ge A^{-|\alpha |-2d-2} M^{(\kappa )}_{\alpha +2(d+1)e_j}, \end{aligned}$$

for $A:=(\max \{A_1,\ldots ,A_{2d+2}\})^{2d+2}$ and $\kappa :=\kappa _{2d+2}$. Then we proceed as in the Roumieu case and prove that

$$\begin{aligned} (1+|t|)^{2(d+1)}\exp \omega _{\mathbf {M}^{(\lambda )}}(t) \le B_1'\exp \omega _{\mathbf {M}^{(\kappa )}}(At), \end{aligned}$$

for $$B_1':=\max \{(4d)^{d+1},\max _{|t|\le \sqrt{d}} (1+|t|)^{2(d+1)}\exp \omega _{\mathbf {M}^{(\lambda )}}(t)\}$$. $$\square$$

### Lemma 5

*Let* $\mathcal {M}$ *be a weight matrix that satisfies* (3.7). *Then*

3.14 $$\begin{aligned} \begin{aligned}&\forall \;\lambda >0, N\in {\mathbb {N}}\ \exists \;0<\kappa \le \lambda , A,B\ge 1\ \forall t\in {\mathbb {R}}^d\setminus \{0\}:\\&\omega _{\mathbf {M}^{(\lambda )}}(t)+N\log |t|\le \omega _{\mathbf {M}^{(\kappa )}}(At)+B. \end{aligned} \end{aligned}$$

*Let* $\mathcal {M}$ *be a weight matrix that satisfies* (3.6). *Then*

3.15 $$\begin{aligned} \begin{aligned}&\forall \;\lambda >0, N\in {\mathbb {N}}\ \exists \;\kappa \ge \lambda , A,B\ge 1\ \forall t\in {\mathbb {R}}^d\setminus \{0\}: \\&\omega _{\mathbf {M}^{(\kappa )}}(t)+N\log |t|\le \omega _{\mathbf {M}^{(\lambda )}}(At)+B. \end{aligned} \end{aligned}$$

### Proof

If $t\in {\mathbb {R}}^d\setminus \{0\}$, then by the definition of the associated weight function, for $1\le j\le d$ such that $|t|_\infty =t_j$,

3.16 $$\begin{aligned} |t|^N\exp \omega _{\mathbf {M}^{(\lambda )}}(t)\le&(\sqrt{d}|t|_\infty )^N\exp \omega _{\mathbf {M}^{(\lambda )}}(t) =d^{N/2}|t_j|^N\exp \omega _{\mathbf {M}^{(\lambda )}}(t)\nonumber \\ =&d^{N/2}|t^{Ne_j}|\sup _{\alpha \in {\mathbb {N}}_{0,t}^d}\frac{|t^\alpha |}{M^{(\lambda )}_\alpha } =d^{N/2} \sup _{\alpha \in {\mathbb {N}}_{0,t}^d}\frac{|t^{\alpha +Ne_j}|}{M^{(\lambda )}_\alpha }, \end{aligned}$$

where ${\mathbb {N}}^d_{0,t}$ is defined by (2.9). This estimate is valid for any given index $\lambda >0$.

In the Beurling case, by *N* iterated applications of (3.7) we find $\kappa _N\le \kappa _{N-1}\le \ldots \le \kappa _1\le \lambda$ and $A_1,\ldots ,A_N\ge 1$ such that, for $A:=(\max \{A_1,\ldots ,A_N\})^N$ and $\kappa :=\kappa _N$, we have, proceeding as in (3.13), $M^{(\kappa )}_{\alpha +Ne_j}\le A^{|\alpha |+N} M^{(\lambda )}_\alpha .$ Therefore,

$$\begin{aligned} |t|^N\exp \omega _{\mathbf {M}^{(\lambda )}}(t) \le d^{N/2} \sup _{\alpha \in {\mathbb {N}}_{0,t}^d} \frac{|(At)^{\alpha +Ne_j}|}{M^{(\kappa )}_{\alpha +Ne_j}} \le d^{N/2}\exp \omega _{\mathbf {M}^{(\kappa )}}(At), \end{aligned}$$

and we conclude that (3.14) is satisfied for $B:=\max \{\frac{N}{2}\log d,1\}$.

In the Roumieu case, we make *N* iterated applications of (3.6) and we find indices $\kappa :=\kappa _N\ge \kappa _{N-1}\ge \ldots \ge \kappa _1\ge \lambda$ and $A_1,\ldots ,A_N\ge 1$ such that, for $A:=(\max \{A_1,\ldots ,A_N\})^N$ and $\kappa =\kappa _N$, as in (3.12) we have that $M^{(\lambda )}_{\alpha +Ne_j}\le A^{|\alpha |+N}M^{(\kappa )}_\alpha$ and hence from (3.16):

$$\begin{aligned} |t|^N\exp \omega _{\mathbf {M}^{(\kappa )}}(t)\le&d^{N/2} \sup _{\alpha \in {\mathbb {N}}_{0,t}^d} \frac{|t^{\alpha +Ne_j}|}{M^{(\kappa )}_{\alpha }} \le d^{N/2} \sup _{\alpha \in {\mathbb {N}}_{0,t}^d} \frac{|(At)^{\alpha +Ne_j}|}{M^{(\lambda )}_{\alpha +Ne_j}}\\ \le&d^{N/2}\exp \omega _{\mathbf {M}^{(\lambda )}}(At), \end{aligned}$$

so that (3.15) is satisfied with $B=\max \{\frac{N}{2}\log d,1\}$.$\square$

Now, we consider the different system of seminorms

3.17 $$\begin{aligned} \Vert f\Vert _{2,\mathbf{M}^{(\lambda )},h}:=\sup _{\alpha ,\beta \in {\mathbb {N}}_0^d} \frac{\Vert x^\alpha \partial ^\beta f\Vert _2}{h^{|\alpha +\beta |}M^{(\lambda )}_{\alpha +\beta }}, \qquad \lambda ,h>0, \end{aligned}$$

on $\mathcal {S}_{(\mathcal {M})}$ and $\mathcal {S}_{\{\mathcal {M}\}}$, where $\Vert \cdot \Vert _2$ is the $L^2$ norm. Under suitable conditions on the weight matrix $\mathcal {M}$, it turns out to be equivalent to the previous one given by sup norms, as we prove in the following:

### Proposition 1

*Let* $\mathcal {M}$ *be a weight matrix as defined in* (3.1) *that satisfies* (3.3) *and* (3.7) ((3.2) *and* (3.6)). *Then the system of seminorms* $\Vert \cdot \Vert _{\infty ,\mathbf{M}^{(\lambda )},h}$ in $\mathcal {S}_{(\mathcal {M})}$ ($\mathcal {S}_{\{\mathcal {M}\}}$) *is equivalent to the system of seminorms* $\Vert \cdot \Vert _{2,\mathbf{M}^{(\lambda )},h}$. *More precisely, in the Beurling case we have the following two* *conditions for every* $f\in C^\infty ({\mathbb {R}}^d)$:

3.18 $$\begin{aligned}&\begin{aligned}&\exists \;C_1>0\ \forall \, \lambda ,h>0\ \exists \;\kappa>0, {{\tilde{h}}}=\tilde{h}_{\lambda ,h}>0:\\&\Vert f\Vert _{2,\mathbf{M}^{(\lambda )},h}\le C_1\Vert f\Vert _{\infty ,\mathbf{M}^{(\kappa )},{\tilde{h}}}, \end{aligned}\end{aligned}$$

3.19 $$\begin{aligned}&\begin{aligned}&\forall \,\lambda ,h>0\ \exists \;\widetilde{\kappa }>0, C_{\lambda ,h}>0,{\tilde{h}}={\tilde{h}}_{\lambda ,h}>0:\\&\Vert f\Vert _{\infty ,\mathbf{M}^{(\lambda )},h}\le C_{\lambda ,h}\Vert f\Vert _{2,\mathbf{M}^{(\widetilde{\kappa })},{\tilde{h}}}\,; \end{aligned} \end{aligned}$$

*in the Roumieu case we have the following two conditions, for every* $f\in C^\infty ({\mathbb {R}}^d)$,

3.20 $$\begin{aligned}&\begin{aligned}&\forall \;\lambda ,h>0\,\exists \;C_{\lambda ,h}>0,\, \exists \;\kappa \ge \lambda ,{\tilde{h}}>0:\\&\Vert f\Vert _{2,\mathbf{M}^{(\kappa )},{\tilde{h}}}\le C_{\lambda ,h}\Vert f\Vert _{\infty ,\mathbf{M}^{(\lambda )},h}, \end{aligned}\end{aligned}$$

3.21 $$\begin{aligned}&\begin{aligned}&\forall \,\lambda ,h>0\ \exists \, C_{\lambda ,h}>0, \widetilde{\kappa }>0,{\tilde{h}}>0:\\&\Vert f\Vert _{\infty ,\mathbf{M}^{(\widetilde{\kappa })},{\tilde{h}}}\le C_{\lambda ,h}\Vert f\Vert _{2,\mathbf{M}^{(\lambda )},h}\,. \end{aligned} \end{aligned}$$

### Proof

Let $f\in C^\infty ({\mathbb {R}}^d)$. Then for $C_1=(\int _{{\mathbb {R}}^d}\frac{1}{(1+|x|^2)^{d+1}}dx)^{1/2}$, we have

$$\begin{aligned} \Vert x^\alpha \partial ^\beta f\Vert _2 \le C_1\Vert (1+|x|^2)^{\frac{d+1}{2}}x^\alpha \partial ^\beta f(x)\Vert _\infty . \end{aligned}$$

If $$|x|_\infty \le 1$$, then

$$\begin{aligned} (1+|x|^2)^{\frac{d+1}{2}}\le (1+d|x|_\infty ^2)^{\frac{d+1}{2}} \le (1+d)^{\frac{d+1}{2}}. \end{aligned}$$

On the other hand, if $|x|_\infty \ge 1$ then

$$\begin{aligned} (1+|x|^2)^{\frac{d+1}{2}}\le (|x|_\infty ^2+|x|^2)^{\frac{d+1}{2}} \le (|x|_\infty ^2+d|x|_\infty ^2)^{\frac{d+1}{2}} \le (d+1)^{\frac{d+1}{2}}|x|_\infty ^{d+1}. \end{aligned}$$

Therefore, for any fixed $x\in {\mathbb {R}}^d$, being $|x|_\infty =|x_j|$ for some $1\le j\le d$, we have

$$\begin{aligned} |(1+|x|^2)^{\frac{d+1}{2}}x^\alpha |\le (d+1)^{\frac{d+1}{2}} \max \{|x^{\alpha +(d+1)e_j}|,|x^\alpha |\} \end{aligned}$$

and hence

3.22 $$\begin{aligned} \begin{aligned} \Vert x^\alpha \partial ^\beta f\Vert _2\le C_1(d+1)^{\frac{d+1}{2}} \max \{&\Vert x^{\alpha +(d+1)e_1}\partial ^\beta f\Vert _\infty , \Vert x^{\alpha +(d+1)e_2}\partial ^\beta f\Vert _\infty ,\\&\ldots ,\Vert x^{\alpha +(d+1)e_d}\partial ^\beta f\Vert _\infty ,\Vert x^\alpha \partial ^\beta f\Vert _\infty \}. \end{aligned} \end{aligned}$$

Now, we consider separately the Beurling and Roumieu cases. In the Beurling case, for every $\lambda ,h>0$, we first estimate $\Vert x^{\alpha +(d+1)e_j} \partial ^\beta f\Vert _{2,\mathbf{M}^{(\lambda )},h}$ to use (3.22). By $(d+1)$ iterated applications of (3.7) there exist $0<\kappa :=\kappa _{d+1}\le \kappa _d\le \ldots \le \kappa _1\le \lambda$ and $A_1,\ldots ,A_{d+1}\ge 1$ ($A_j$ depending on $\lambda$) such that, proceeding as in (3.13), we obtain $M^{(\kappa )}_{\alpha +\beta +(d+1)e_j}\le A_{\lambda }^{|\alpha +\beta |+d+1} M^{(\lambda )}_{\alpha +\beta }$ for $A_\lambda =(\max \{A_1,\ldots ,A_{d+1}\})^{d+1}\ge 1$. Hence, we deduce

$$\begin{aligned} \frac{\Vert x^{\alpha +(d+1)e_j}\partial ^\beta f\Vert _\infty }{h^{|\alpha +\beta |}M^{(\lambda )}_{\alpha +\beta }}\le \frac{\Vert x^{\alpha +(d+1)e_j}\partial ^\beta f\Vert _\infty }{h^{|\alpha +\beta |+d+1} M^{(\kappa )}_{\alpha +\beta +(d+1)e_j}}\cdot h^{d+1}A_{\lambda }^{|\alpha +\beta |+d+1}. \end{aligned}$$

Therefore, from (3.22) and the fact that $\mathbf{M}^{(\kappa )}\le \mathbf{M}^{(\lambda )}$, we have for every $\lambda ,h>0$,

3.23 $$\begin{aligned} \begin{aligned} \frac{\Vert x^\alpha \partial ^\beta f\Vert _2}{h^{|\alpha +\beta |}M^{(\lambda )}_{\alpha +\beta }} \le&C_1(d+1)^{\frac{d+1}{2}} \max \bigg \{\frac{\Vert x^{\alpha +(d+1)e_1}\partial ^\beta f\Vert _\infty }{h^{|\alpha +\beta |+d+1}M^{(\kappa )}_{\alpha +\beta +(d+1)e_1}} h^{d+1}A_{\lambda }^{|\alpha +\beta |+d+1},\\&\ldots ,\frac{\Vert x^{\alpha +(d+1)e_d}\partial ^\beta f\Vert _\infty }{h^{|\alpha +\beta |+d+1}M^{(\kappa )}_{\alpha +\beta +(d+1)e_d}} h^{d+1}A_{\lambda }^{|\alpha +\beta |+d+1}, \frac{\Vert x^\alpha \partial ^\beta f\Vert _\infty }{h^{|\alpha +\beta |}M^{(\kappa )}_{\alpha +\beta }} \bigg \}. \end{aligned} \end{aligned}$$

If $$h\ge 1$$ then $$h^{d+1}A_\lambda ^{|\alpha +\beta |+d+1}\le (hA_\lambda )^{|\alpha +\beta |+d+1}$$.

If $0<h<1$ then $h^{d+1}A_\lambda ^{|\alpha +\beta |+d+1}\le A_\lambda ^{|\alpha +\beta |+d+1}$. Hence, for

$$\begin{aligned} {\tilde{h}}:={\left\{ \begin{array}{ll} \min \left\{ \frac{1}{A_\lambda },h\right\} =\frac{1}{A_\lambda },&{}\quad \text{ if }\ h\ge 1,\\ \min \left\{ \frac{h}{A_\lambda },h\right\} =\frac{h}{A_\lambda },&{}\quad \text{ if }\ 0<h<1, \end{array}\right. } \end{aligned}$$

we obtain

$$\begin{aligned} \Vert f\Vert _{2,\mathbf{M}^{(\lambda )},h}\le C_1(d+1)^{\frac{d+1}{2}}\Vert f\Vert _{\infty ,\mathbf{M}^{(\kappa )},{\tilde{h}}}. \end{aligned}$$

This shows (3.18).

Now, since $\delta !\le \delta _1^{\delta _1}\dots \delta _d^{\delta _d}=\delta ^\delta$, we have $$\frac{\alpha !}{(\alpha -\delta )!}\le \left( {\begin{array}{c}\alpha \\ \delta \end{array}}\right) \delta !\le 2^{|\alpha |}\delta ^{\delta }.$$ So it follows by Leibniz’s rule and [28, formula (2.3)] that, for some $C_2>0$,

3.24 $$\begin{aligned} \Vert x^\alpha \partial ^\beta f\Vert _\infty \le&C_2\sup _{|\gamma |_\infty \le 2d+2} \Vert \partial ^\gamma (x^\alpha \partial ^\beta f)\Vert _2\nonumber \\ \le&C_2\sup _{|\gamma |_\infty \le 2d+2} \sum _{\delta \le \gamma }\left( {\begin{array}{c}\gamma \\ \delta \end{array}}\right) \Vert (\partial ^\delta x^\alpha )\partial ^{\beta +\gamma -\delta }f\Vert _2\nonumber \\ \le&C_2 \sup _{|\gamma |_\infty \le 2d+2} \sum _{\genfrac{}{}{0.0pt}1{\delta \le \gamma }{\delta \le \alpha }}\left( {\begin{array}{c}\gamma \\ \delta \end{array}}\right) 2^{|\alpha |}\delta ^{\delta } \Vert x^{\alpha -\delta }\partial ^{\beta +\gamma -\delta }f\Vert _2\,. \end{aligned}$$

On the other hand, by $|\gamma |$ iterated applications of (3.7), there exist $0<\kappa :=\kappa _{|\gamma |}\le \kappa _{|\gamma |-1}\le \ldots \le \kappa _1\le \lambda$ and $A_1,\ldots ,A_{|\gamma |}\ge 1$ such that, for $A_\lambda :=\max \limits _{\vert \gamma \vert _\infty \le 2d+2}(\max \{A_1,\ldots ,A_{|\gamma |}\})^{|\gamma |}$, we have $M^{(\kappa )}_{\alpha +\beta +\gamma }\le A_\lambda ^{|\alpha +\beta +\gamma |} M^{(\lambda )}_{\alpha +\beta }$. By (3.3), there exist $0<\widetilde{\kappa }\le \kappa$ and $H>0$ such that for all $C>0$ there is $B>0$ so that

$$\begin{aligned} \frac{\Vert x^\alpha \partial ^\beta f\Vert _\infty }{h^{|\alpha +\beta |}M^{(\lambda )}_{\alpha +\beta }}\le&C_2 \sup _{|\gamma |_\infty \le 2d+2} \sum _{\genfrac{}{}{0.0pt}1{\delta \le \gamma }{\delta \le \alpha }}\left( {\begin{array}{c}\gamma \\ \delta \end{array}}\right) \frac{\Vert x^{\alpha -\delta }\partial ^{\beta +\gamma -\delta }f\Vert _2}{h^{|\alpha +\beta +\gamma -2\delta |} M^{(\widetilde{\kappa })}_{\alpha +\beta +\gamma -2\delta }}\cdot h^{|\gamma -2\delta |}\\&\cdot 2^{|\alpha |}A_\lambda ^{|\alpha +\beta +\gamma |} BC^{|2\delta |}H^{|\alpha +\beta +\gamma |}. \end{aligned}$$

Observe that $\widetilde{\kappa }$ may depend on $\gamma$. From (3.1) we can consider in the previous estimates, instead of $\widetilde{\kappa }$, the minimum of all these $\widetilde{\kappa }$ for $\vert \gamma \vert _\infty \le 2d+2$, so that we can finally choose $\widetilde{\kappa }$ independent of $\gamma$. Since $|\gamma |\le d|\gamma |_\infty \le 2d(d+1)$ we have

$$\begin{aligned} \frac{\Vert x^\alpha \partial ^\beta f\Vert _\infty }{h^{|\alpha +\beta |}M^{(\lambda )}_{\alpha +\beta }} \le&C_2B(2CHA_\lambda )^{4d(d+1)}\\&\cdot \sup _{\vert \gamma \vert _\infty \le 2d+2} \sum _{\genfrac{}{}{0.0pt}1{\delta \le \gamma }{\delta \le \alpha }}\left( {\begin{array}{c}\gamma \\ \delta \end{array}}\right) \frac{\Vert x^{\alpha -\delta }\partial ^{\beta +\gamma -\delta }f\Vert _2}{h^{|\alpha +\beta +\gamma -2\delta |} M^{(\widetilde{\kappa })}_{\alpha +\beta +\gamma -2\delta }}\\&\cdot (2HA_\lambda )^{|\alpha +\beta +\gamma -2\delta |} h^{|\gamma -2\delta |}. \end{aligned}$$

Now, if $h\ge 1$, then $h^{|\gamma -2\delta |}\le h^{|\alpha +\beta +\gamma -2\delta |}$. And if $0<h<1$, then $h^{|\gamma -2\delta |}\le 1$ when $|\gamma -2\delta |\ge 0$ and $h^{|\gamma -2\delta |}\le h^{-|\gamma |} \le h^{-2d(d+1)}$ when $|\gamma -2\delta |<0$. For

3.25 $$\begin{aligned} {\tilde{h}}={\left\{ \begin{array}{ll} \frac{1}{2HA_\lambda }&{}\text{ if }\ h\ge 1\\ \frac{h}{2HA_\lambda }&{}\text{ if }\ 0<h<1, \end{array}\right. } \end{aligned}$$

taking into account that

$$\begin{aligned} \sum _{\delta \le \gamma }\left( {\begin{array}{c}\gamma \\ \delta \end{array}}\right) \le d^{|\gamma |}\le d^{2d(d+1)}, \end{aligned}$$

we finally have that for all $\lambda ,h>0$ there exist $\widetilde{\kappa }$, $C_{\lambda ,h}>0$ and ${\tilde{h}}>0$, such that

3.26 $$\begin{aligned} \Vert f\Vert _{\infty ,\mathbf{M}^{(\lambda )},h}\le C_{\lambda ,h}\Vert f\Vert _{2,\mathbf{M}^{(\widetilde{\kappa })},{\tilde{h}}}. \end{aligned}$$

Since neither *H* nor $A_{\lambda }$ are depending on *h*, we have $\tilde{h}\rightarrow 0$ as $h\rightarrow 0$. This shows (3.19) and concludes the proof in the Beurling case.

Let us now consider the Roumieu case. In (3.22), for any given $\lambda$ by $(d+1)$ iterated applications of (3.6), we obtain $\kappa :=\kappa _{d+1}\ge \kappa _d\ge \ldots \ge \kappa _1\ge \lambda >0$ and $A_1,\ldots ,A_{d+1}\ge 1$ such that, for $A_\lambda :=(\max \{A_1,\ldots ,A_{d+1}\})^{d+1}$, we have $M^{(\lambda )}_{\alpha +\beta +(d+1)e_j}\le A_\lambda ^{|\alpha +\beta |+d+1}M^{(\kappa )}_{\alpha +\beta }$. Then from (3.22) and the fact that $M^{(\kappa )}_{\alpha +\beta }\ge M^{(\lambda )}_{\alpha +\beta }$ we obtain, given a fixed $h>0$, for $\tilde{h}:=\max \{ hA_\lambda ,1\}$,

$$\begin{aligned} \frac{\Vert x^\alpha \partial ^\beta f\Vert _2}{M^{(\kappa )}_{\alpha +\beta }}\le&C_1 (d+1)^{\frac{d+1}{2}} \max \Biggr \{ \frac{\Vert x^\alpha \partial ^\beta f\Vert _\infty }{M^{(\kappa )}_{\alpha +\beta }},\ \frac{\Vert x^{\alpha +(d+1)e_j}\partial ^\beta f\Vert _\infty }{M^{(\lambda )}_{\alpha +\beta +(d+1)e_j}} A_\lambda ^{\vert \alpha +\beta \vert +d+1}\Biggr \} \\ \le&C_1 (d+1)^{\frac{d+1}{2}} \tilde{h}^{\vert \alpha +\beta \vert +d+1}\\&\cdot \max \Biggr \{ \frac{\Vert x^\alpha \partial ^\beta f\Vert _\infty }{h^{\vert \alpha +\beta \vert }M^{(\lambda )}_{\alpha +\beta }},\ \frac{\Vert x^{\alpha +(d+1)e_j}\partial ^\beta f\Vert _\infty }{h^{\vert \alpha +\beta \vert +d+1}M^{(\lambda )}_{\alpha +\beta +(d+1)e_j}} \Biggr \}. \end{aligned}$$

Hence, dividing by $\tilde{h}^{\vert \alpha +\beta \vert }$,

$$\begin{aligned} \frac{\Vert x^\alpha \partial ^\beta f\Vert _2}{\tilde{h}^{\vert \alpha +\beta \vert }M^{(\kappa )}_{\alpha +\beta }}\le C_1 (d+1)^{\frac{d+1}{2}} \tilde{h}^{d+1} \Vert f\Vert _{\infty ,\mathbf{M}^{(\lambda )},h}; \end{aligned}$$

then (3.20) is proved, with $C_{\lambda ,h}=C_1 (d+1)^{\frac{d+1}{2}} \tilde{h}^{d+1}$ (observe that $\tilde{h}$ depends on *h* and $\lambda$).

Now, given any $\lambda >0$ consider $\kappa \ge \lambda >0$ and $B,C,H>0$ as in (3.2). Then, by $|\gamma |$ iterated applications of (3.6), there exist $\widetilde{\kappa }:=\kappa _{|\gamma |}\ge \ldots \ge \kappa _1\ge \kappa \ge \lambda$ and $A_1,\ldots ,A_{|\gamma |}\ge 1$ such that, for $A_\lambda :=(\max \{A_1,\ldots ,A_{|\gamma |}\})^{|\gamma |}$, $M^{(\kappa )}_{\alpha +\beta +\gamma }\le A_\lambda ^{|\alpha +\beta +\gamma |}M^{({\tilde{\kappa }})}_{\alpha +\beta }.$ So, from (3.24) with $h=1$ and ${\tilde{\kappa }}$ instead of $\lambda$, applying (3.2) and proceeding as before, we get

3.27 $$\begin{aligned} \begin{aligned} \frac{\Vert x^\alpha \partial ^\beta f\Vert _\infty }{M^{({\tilde{\kappa }})}_{\alpha +\beta }}\le&C_2 BC^{4d(d+1)}\\&\cdot \sup _{|\gamma |_\infty \le 2d+2} \sum _{\genfrac{}{}{0.0pt}1{\delta \le \gamma }{\delta \le \alpha }}\left( {\begin{array}{c}\gamma \\ \delta \end{array}}\right) (2A_\lambda H)^{|\alpha +\beta +\gamma |} \frac{\Vert x^{\alpha -\delta }\partial ^{\beta +\gamma -\delta }f\Vert _2}{M^{(\lambda )}_{\alpha +\beta +\gamma -2\delta }}\,. \end{aligned} \end{aligned}$$

Since for every $h>0$ and $\alpha ,\beta ,\gamma ,\delta$ as above

$$\begin{aligned} \frac{\Vert x^{\alpha -\delta }\partial ^{\beta +\gamma -\delta }f\Vert _2}{h^{\vert \alpha +\beta +\gamma -2\delta \vert } M^{(\lambda )}_{\alpha +\beta +\gamma -2\delta }}\le \Vert f\Vert _{2,\mathbf{M}^{(\lambda )},h}\,, \end{aligned}$$

dividing (3.27) by $(2A_\lambda Hh)^{\vert \alpha +\beta \vert }$ we obtain

$$\begin{aligned} \frac{\Vert x^\alpha \partial ^\beta f\Vert _{\infty }}{(2A_\lambda Hh)^{\vert \alpha +\beta \vert } M^{(\widetilde{\kappa })}_{\alpha +\beta }}\le&\Vert f\Vert _{2,\mathbf{M}^{(\lambda )},h}\, C_2 BC^{4d(d+1)}\\&\cdot \sup _{|\gamma |_\infty \le 2d+2} \sum _{\delta \le \gamma }\left( {\begin{array}{c}\gamma \\ \delta \end{array}}\right) (2A_\lambda Hh)^{|\gamma |} h^{-\vert 2\delta \vert }. \end{aligned}$$

Taking the $\sup$ on $\alpha$ and $\beta$ in the left-hand side, we then get (3.21) with $\tilde{h}=2A_\lambda Hh$ and

$$\begin{aligned} C_{\lambda ,h}=C_2 BC^{4d(d+1)} \sup _{|\gamma |_\infty \le 2d+2} \sum _{\delta \le \gamma }\left( {\begin{array}{c}\gamma \\ \delta \end{array}}\right) (2A_\lambda Hh)^{|\gamma |} h^{-\vert 2\delta \vert }. \end{aligned}$$

$$\square$$

We observe that in (3.18) the constant $C_1$ is fixed (it depends only on the dimension d), and moreover, we only need (3.7) to prove it. On the other hand, to obtain (3.19) we consider (3.7) and (3.3). In the Roumieu case, we just need (3.6) to prove (3.20), while for the proof of (3.21) we use (3.2) to choose $\kappa \ge \lambda$ and then (3.6) to get ${\tilde{\kappa }}\ge \kappa$.

## Hermite functions: properties in the matrix setting

We recall the definition of the *Hermite functions* $H_\gamma$ for $\gamma \in {\mathbb {N}}_0^d$:

$$\begin{aligned} H_\gamma (x):=(2^{|\gamma |}\gamma !\pi ^{d/2})^{-1/2}h_\gamma (x) \exp \left( -\sum _{j=1}^d\frac{x_j^2}{2}\right) ,\qquad x\in {\mathbb {R}}^d, \end{aligned}$$

where $h_\gamma$ are the Hermite polynomials

$$\begin{aligned} h_\gamma (x):=(-1)^{|\gamma |}\exp \left( \sum _{j=1}^dx_j^2\right) \cdot \partial ^\gamma \exp \left( -\sum _{j=1}^dx_j^2\right) ,\qquad x\in {\mathbb {R}}^d. \end{aligned}$$

As in [28] we consider, for $f\in C^\infty ({\mathbb {R}}^d)$, the operators

$$\begin{aligned}&A_{\pm ,i}(f):=\mp \partial _{x_i}f+x_i f,\qquad 1\le i\le d,\\&A_{\pm }^\alpha (f):=\prod _{i=1}^dA_{\pm ,i}^{\alpha _i}(f),\qquad \alpha \in {\mathbb {N}}_0^d, \end{aligned}$$

with $$A_{\pm ,i}^0:=\mathop {\mathrm{id}}\nolimits$$.

By [32, Example 29.5(2)], setting $H_\beta =0$ if $\beta _j=-1$ for some $1\le j\le d$, we have

$$\begin{aligned} A_{-,j}(H_\gamma )=\sqrt{2\gamma _j}H_{\gamma -e_j},\quad \gamma \in {\mathbb {N}}_0^d. \end{aligned}$$

It follows that, for $\alpha ,\gamma \in {\mathbb {N}}_0^d$,

4.1 $$\begin{aligned} \ A_{-}^\alpha (H_{\gamma +\alpha })=\prod _{1\le j\le d}A_{-,j}^{\alpha _j}(H_{\gamma +\alpha }) =\prod _{1\le j\le d}(\sqrt{2\gamma _j})^{\alpha _j}H_\gamma =\sqrt{2^{|\alpha |}\gamma ^\alpha }H_\gamma . \end{aligned}$$

We also recall the following two lemmas from [28]:

### Lemma 6

*Let* $f\in C^\infty ({\mathbb {R}}^d)$. *Then, for all* $\gamma \in {\mathbb {N}}_0^d$ *and* $x\in {\mathbb {R}}^d$,

$$\begin{aligned} (A_+^\gamma f)(x)=\sum _{\alpha +\beta \le \gamma }C_{\alpha ,\beta }(\gamma ) x^\alpha \partial ^\beta f(x), \end{aligned}$$

*for some coefficients* $C_{\alpha ,\beta }(\gamma )$ *satisfying*

$$\begin{aligned} |C_{\alpha ,\beta }(\gamma )|\le 3^{|\gamma |}\left( \frac{\gamma !}{(\alpha +\beta )!}\right) ^{1/2} \!\!\!,\qquad \alpha ,\beta ,\gamma \in {\mathbb {N}}_0^d. \end{aligned}$$

### Lemma 7

*For all* $$\alpha ,\beta ,\gamma \in {\mathbb {N}}_0^d$$

$$\begin{aligned} \Vert x^\alpha \partial ^\beta H_\gamma \Vert _2\le 2^{\frac{|\alpha +\beta |}{2}} \left( \frac{(\alpha +\beta +\gamma )!}{\gamma !}\right) ^{1/2}. \end{aligned}$$

We can generalize Lemma 3.1(b) of [28] in the following way:

### Lemma 8

*Let* $\mathbf {M}=(M_\alpha )_{\alpha \in {\mathbb {N}}_0^d}$ *and* $\mathbf {N}=(N_\alpha )_{\alpha \in {\mathbb {N}}_0^d}$ *be two sequences satisfying* (2.12) *for some* $C,B,H>0$. *Assume that* $f\in C^\infty ({\mathbb {R}}^d)$ *satisfies, for some* $C_1>0$ *and for the same constant* *C* *as in* (2.12),

4.2 $$\begin{aligned} \Vert f\Vert _{2,\mathbf {M},C}=\sup _{\alpha ,\beta \in {\mathbb {N}}_0^d}\frac{\Vert x^\alpha \partial ^\beta f\Vert _2}{C^{|\alpha +\beta |}M_{\alpha +\beta }}\le C_1. \end{aligned}$$

*Then*

$$\begin{aligned} \Vert A_+^\gamma f\Vert _2\le C_1Be^{d/2}(9\sqrt{2}HC)^{|\gamma |}N_\gamma , \quad \gamma \in {\mathbb {N}}_0^d. \end{aligned}$$

### Proof

By Stirling’s inequality $e\left( \frac{n}{e}\right) ^n\le n!\le en\left( \frac{n}{e}\right) ^n$ for any $n\in {\mathbb {N}}$. Hence, by Lemma 6 and assumption (4.2), we have

$$\begin{aligned} \Vert A_+^\gamma f\Vert _2\le&\sum _{\alpha +\beta \le \gamma }|C_{\alpha ,\beta }(\gamma )|\cdot \Vert x^\alpha \partial ^\beta f\Vert _2\\ \le&C_13^{|\gamma |}\sum _{\alpha +\beta \le \gamma }\left( {\begin{array}{c}\gamma \\ \alpha +\beta \end{array}}\right) ^{1/2} (\gamma -\alpha -\beta )!^{1/2}C^{|\alpha +\beta |}M_{\alpha +\beta }\\ \le&C_13^{|\gamma |}\sum _{\alpha +\beta \le \gamma }\left( {\begin{array}{c}\gamma \\ \alpha +\beta \end{array}}\right) ^{1/2} e^{d/2}\left( \prod _{j=1}^d(\gamma _j-\alpha _j-\beta _j)^{1/2}\right) \\&\cdot \frac{(\gamma -\alpha -\beta )^{\frac{\gamma -\alpha -\beta }{2}}}{\exp \left\{ \frac{\vert \gamma -\alpha -\beta \vert }{2}\right\} } C^{|\alpha +\beta |} M_{\alpha +\beta }\\ \le&C_13^{|\gamma |}2^{|\gamma |/2}e^{d/2} \sum _{\alpha +\beta \le \gamma }\left( {\begin{array}{c}\gamma \\ \alpha +\beta \end{array}}\right) (\gamma -\alpha -\beta )^{\frac{\gamma -\alpha -\beta }{2}}C^{|\alpha +\beta |} M_{\alpha +\beta }. \end{aligned}$$

Applying now (2.12) and $$\sum _{\alpha +\beta \le \gamma } \left( {\begin{array}{c}\gamma \\ \alpha +\beta \end{array}}\right) \le 3^{|\gamma |}$$ (by [28, pg 274]), we get

$$\begin{aligned} \Vert A_+^{\gamma } f\Vert _2\le&C_1 e^{d/2}(3\sqrt{2})^{|\gamma |} \sum _{\alpha +\beta \le \gamma }\left( {\begin{array}{c}\gamma \\ \alpha +\beta \end{array}}\right) BC^{|\gamma -\alpha -\beta |}H^{|\gamma |}N_\gamma C^{|\alpha +\beta |}\\ \le&C_1B e^{d/2}(9\sqrt{2})^{|\gamma |}(CH)^{|\gamma |}N_\gamma . \end{aligned}$$

$$\square$$

As a corollary, we immediately have the following:

### Lemma 9

*Let* $\mathcal {M}$ *be a weight matrix satisfying* (3.2) *and assume that* $f\in C^\infty ({\mathbb {R}}^d)$ *satisfies, for some* $\lambda ,C_1>0$

4.3 $$\begin{aligned} \Vert f\Vert _{2,\mathbf {M}^{(\lambda )},C}\le C_1 \end{aligned}$$

*for the constant* *C* *of* (3.2). *Then*

$$\begin{aligned} \Vert A_+^\gamma f\Vert _2\le C_1B e^{d/2}(9\sqrt{2}HC)^{|\gamma |}M^{(\kappa )}_\gamma , \qquad \forall \gamma \in {\mathbb {N}}_0^d, \end{aligned}$$

*with* $\kappa ,B,H,C$ *as in* (3.2).

*If* $\mathcal {M}$ *satisfies* (3.3) *and if, for some* $\lambda >0$, $f\in C^\infty ({\mathbb {R}}^d)$ *satisfies*

$$\begin{aligned} \Vert f\Vert _{2,\mathbf {M}^{(\kappa )},C}\le C_1 \end{aligned}$$

*for the constant* $\kappa \le \lambda$ *of* (3.3) *and for some* $C,C_1>0$, *then*

$$\begin{aligned} \Vert A_+^\gamma f\Vert _2\le C_1B e^{d/2}(9\sqrt{2}HC)^{|\gamma |}M^{(\lambda )}_\gamma , \qquad \forall \gamma \in {\mathbb {N}}_0^d, \end{aligned}$$

*where* $H=H(\lambda )$ *and* $B=B(C,\lambda )$ *are given by* (3.3).

The following lemma generalizes [28, Lemma 3.2(b)].

### Lemma 10

*Let* $\mathbf {M}=(M_\alpha )_{\alpha \in {\mathbb {N}}_0^d}$ *and* $\mathbf {N}=(N_\alpha )_{\alpha \in {\mathbb {N}}_0^d}$ *be two weight sequences satisfying* (2.12). *Then*

$$\begin{aligned} \Vert H_\gamma \Vert _{2,\mathbf {N},2HC}= \sup _{\alpha ,\beta \in {\mathbb {N}}_0^d}\frac{\Vert x^\alpha \partial ^\beta H_\gamma \Vert _2}{(2HC)^{|\alpha +\beta |}N_{\alpha +\beta }}\le Be^{\omega _\mathbf {M}(\gamma ^{1/2}/C)}, \qquad \forall \gamma \in {\mathbb {N}}_0^d, \end{aligned}$$

*where* $\gamma ^{1/2}:=(\gamma _1^{1/2},\ldots ,\gamma _d^{1/2})$ *and* $B,C,H>0$ *are the constants in *(2.12).

### Proof

For $\alpha ,\beta ,\gamma \in {\mathbb {N}}_0^d$ we set

$$\begin{aligned}&J:=\{j\in {\mathbb {N}}:\ 1\le j\le d,\alpha _j+\beta _j\le \gamma _j\}\\&J^c:=\{j\in {\mathbb {N}}:\ 1\le j\le d,\alpha _j+\beta _j>\gamma _j\}. \end{aligned}$$

Then for any $\delta \in {\mathbb {N}}^d$ we denote

$$\begin{aligned} \delta _J:=\sum _{j\in J}\delta _je_j, \quad \delta _{J^c}:=\sum _{j\in J^c}\delta _je_j, \end{aligned}$$

so that $\delta =\delta _J+\delta _{J^c}$. By Lemma 7 and (2.12), we have

4.4 $$\begin{aligned} \Vert x^\alpha \partial ^\beta H_\gamma \Vert _2\le&2^{\frac{|\alpha +\beta |}{2}} \left( \frac{(\alpha +\beta +\gamma )!}{\gamma !}\right) ^{1/2} \le 2^{\frac{|\alpha +\beta |}{2}}(\alpha +\beta +\gamma )^{\frac{\alpha +\beta }{2}}\nonumber \\ \le&2^{|\alpha +\beta |}(\alpha _{J^c}+\beta _{J^c})^{\frac{\alpha _{J^c}+\beta _{J^c}}{2}}\gamma _J^{\frac{\alpha _J+\beta _J}{2}}\nonumber \\ \le&B(2HC)^{|\alpha +\beta |}N_{\alpha +\beta }\gamma _J^{\frac{\alpha _J+\beta _J}{2}} \frac{1}{M_{\alpha _J+\beta _J}C^{|\alpha _J+\beta _J|}}. \end{aligned}$$

Now, since $\alpha _J$ has the *j*th entry equal to $\alpha _j$ for $j\in J$ and 0 for $j\in J^c$,

4.5 $$\begin{aligned} \gamma _J^{\frac{\alpha _J+\beta _J}{2}}=\prod _{j\in J} \gamma _j^{\frac{\alpha _j+\beta _j}{2}}=\prod _{j\in J} \gamma _j^{\frac{\alpha _j+\beta _j}{2}} \prod _{j\in J^c}\gamma _j^0=\gamma ^{\frac{\alpha _J+\beta _J}{2}}. \end{aligned}$$

Moreover, by Lemma 3,

4.6 $$\begin{aligned} M_{\alpha _J+\beta _J}C^{|\alpha _J+\beta _J|}\ge \sup _{t\in {\mathbb {R}}^d} |t^{\alpha _J+\beta _J}e^{-\omega _\mathbf {M}(t/C)}| \ge \gamma ^{\frac{\alpha _J+\beta _J}{2}}e^{-\omega _\mathbf {M}(\gamma ^{1/2}/C)}, \end{aligned}$$

taking $$t=\gamma ^{1/2}$$.

If we replace (4.5) and (4.6) in (4.4) we finally get

$$\begin{aligned} \Vert x^\alpha \partial ^\beta H_\gamma \Vert _2\le B(2HC)^{|\alpha +\beta |} N_{\alpha +\beta }e^{\omega _\mathbf {M}(\gamma ^{1/2}/C)}. \end{aligned}$$

$$\square$$

### Proposition 2

*Let* $\mathcal {M}$ *be a weight matrix that satisfies* (3.2) *and* (3.6) ((3.3) *and* (3.7)). *Then* $H_\gamma \in \mathcal {S}_{\{\mathcal {M}\}}$ ($H_\gamma \in \mathcal {S}_{(\mathcal {M})}$) *for all* $\gamma \in {\mathbb {N}}_0^d$.

### Proof

By Lemma 10, if (3.2) is satisfied, we have

$$\begin{aligned}&\forall \lambda>0\,\exists \;\kappa \ge \lambda ,\,\exists B,C,H>0:\\&\Vert x^\alpha \partial ^\beta H_\gamma \Vert _2\le B(2HC)^{|\alpha +\beta |}M^{(\kappa )}_{|\alpha +\beta |} e^{\omega _{\mathbf {M}^{(\lambda )}}(\gamma ^{1/2}/C)}. \end{aligned}$$

Hence, $H_\gamma \in \mathcal {S}_{\{\mathcal {M}\}}$ by Proposition 1. Similarly, in the Beurling case, if (3.3) is satisfied, we obtain

$$\begin{aligned}&\forall \lambda>0\,\exists \;0<\kappa \le \lambda ,\,\exists H>0:\, \forall C>0\,\exists B>0:\\&\Vert x^\alpha \partial ^\beta H_\gamma \Vert _2\le B(2HC)^{|\alpha +\beta |}M^{(\lambda )}_{|\alpha +\beta |} e^{\omega _{\mathbf {M}^{(\kappa )}}(\gamma ^{1/2}/C)}. \end{aligned}$$

So $H_\gamma \in \mathcal {S}_{(\mathcal {M})}$ by Proposition 1.$\square$

The next result gives information about the non-triviality of the classes $\mathcal {S}_{\{\mathcal {M}\}}$ and $\mathcal {S}_{(\mathcal {M})}$. Indeed, we characterize when the Hermite functions $H_{\gamma }$ are contained in such classes.

### Proposition 3

*Let* $\mathcal {M}$ *be a weight matrix that satisfies* (3.6), (3.4); *then the following are equivalent*:

(a): $$\exists \lambda>0\,\exists C,C_1>0:\quad \alpha ^{\alpha /2}\le C_1 C^{|\alpha |}M^{(\lambda )}_\alpha ,\quad \forall \alpha \in {\mathbb {N}}_0^d$$;
(b): $$\mathcal {M}$$ *satisfies* (3.2);
(c): $$H_\gamma \in \mathcal {S}_{\{\mathcal {M}\}}$$ *for all* $$\gamma \in {\mathbb {N}}_0^d$$.

*If* $\mathcal {M}$ *satisfies* (3.7), (3.5), *then the following are equivalent*:

(a)′: $$\forall \lambda ,C>0\,\exists C_1>0:\quad \alpha ^{\alpha /2}\le C_1 C^{|\alpha |}M^{(\lambda )}_\alpha ,\quad \forall \alpha \in {\mathbb {N}}_0^d$$;
(b)′: $$\mathcal {M}$$ *satisfies* (3.3);
(c)′: $$H_\gamma \in \mathcal {S}_{(\mathcal {M})}$$ *for all* $$\gamma \in {\mathbb {N}}_0^d$$.

### Proof

The implications (b) $\Rightarrow$ (c) and (b)′ $\Rightarrow$ (c)′ follow from Proposition 2. To see (a) $\Rightarrow$ (b), fix an arbitrary $\mu >0$ and $\lambda$ as in (a). We have

$$\begin{aligned} \alpha ^{\alpha /2}M_\beta ^{(\mu )}\le C_1 C^{\vert \alpha \vert } M_\alpha ^{(\lambda )} M_\beta ^{(\mu )}. \end{aligned}$$

So, for $\nu =\max \{\lambda ,\mu \}$, by (3.1) and (3.4), there exists $\kappa \ge \nu$ and $A\ge 1$ such that

$$\begin{aligned} \alpha ^{\alpha /2}M^{(\mu )}_\beta \le C_1C^{|\alpha |}M^{(\nu )}_\alpha M^{(\nu )}_\beta \le C_1 C^{|\alpha |}A^{|\alpha +\beta |}M^{(\kappa )}_{\alpha +\beta }, \quad \alpha ,\beta \in {\mathbb {N}}_0^d. \end{aligned}$$

Now, we prove (a)′$\Rightarrow$(b)′. For any given $\lambda >0$, let $0<\kappa \le \lambda$ and $A\ge 1$ such that (3.5) holds. By (a)′ applied to this $\kappa$, there is, for any $C>0$, some $C_1>0$ depending on $\kappa$ and *C* such that

$$\begin{aligned} \alpha ^{\alpha /2}M^{(\kappa )}_\beta \le C_1C^{|\alpha |}M^{(\kappa )}_\alpha M^{(\kappa )}_\beta \le C_1 C^{|\alpha |}A^{|\alpha +\beta |}M^{(\lambda )}_{\alpha +\beta }, \quad \alpha ,\beta \in {\mathbb {N}}_0^d. \end{aligned}$$

If (*c*) holds, in particular, $H_0\in \mathcal {S}_{\{\mathcal {M}\}}$. Hence, there exist some $C,h>0$ and $\lambda >0$ such that $\Vert x^{\alpha }H_0\Vert _{\infty }\le Ch^{|\alpha |}M^{(\lambda )}_{\alpha }$ for all $\alpha \in {\mathbb {N}}_0^d$. Taking $x=\alpha ^{1/2}$, $\alpha \in {\mathbb {N}}_0^d$ arbitrary, yields

$$\begin{aligned} |\alpha ^{\alpha /2}H_0(\alpha ^{1/2})|=\frac{1}{\pi ^{d/4}} \alpha _1^{\alpha _1/2}e^{-\alpha _1/2}\cdots \alpha _d^{\alpha _d/2}e^{-\alpha _d/2}=\frac{1}{\pi ^{d/4}} \alpha ^{\alpha /2}e^{-|\alpha |/2}. \end{aligned}$$

Hence, $\alpha ^{\alpha /2}\pi ^{-d/4}e^{-|\alpha |/2}\le \Vert x^{\alpha }H_0\Vert _{\infty }\le Ch^{|\alpha |}M^{(\lambda )}_{\alpha }$ for all $\alpha \in {\mathbb {N}}_0^d$, which shows (a).

The Beurling case (c)′ $\Rightarrow$ (a)′ is analogous since, for any given $\lambda$ and $h>0,$ there exists $C_{\lambda ,h}>0$ such that $\Vert x^{\alpha }H_0\Vert _{\infty }\le C_{\lambda ,h}h^{|\alpha |}M^{(\lambda )}_{\alpha }$ for all $\alpha \in {\mathbb {N}}_0^d$.$\square$

## Matrix sequence spaces

Let us consider, for $\mathbf {M}=(M_\alpha )_{\alpha \in {\mathbb {N}}_0^d}$, the following sequence spaces in the Roumieu and the Beurling cases:

$$\begin{aligned}&\Lambda _{\{\mathbf {M}\}}:=\{\mathbf {c}=(c_\alpha )\in {\mathbb {C}}^{{\mathbb {N}}_0^d}:\ \exists \, h>0,\ \ \Vert \mathbf {c}\Vert _{\mathbf {M},h}:=\sup _{\alpha \in {\mathbb {N}}_0^d}|c_\alpha |e^{\omega _\mathbf {M}(\alpha ^{1/2}/h)}<+\infty \},\\&\Lambda _{(\mathbf {M})}:=\{\mathbf {c}=(c_\alpha )\in {\mathbb {C}}^{{\mathbb {N}}_0^d}:\ \forall \, h>0,\ \ \Vert \mathbf {c}\Vert _{\mathbf {M},h}<+\infty \}. \end{aligned}$$

Since $h\mapsto \omega _\mathbf {M}(\alpha ^{1/2}/h)$ is decreasing we can also write

$$\begin{aligned}&\Lambda _{\{\mathbf {M}\}}=\{\mathbf {c}=(c_\alpha )\in {\mathbb {C}}^{{\mathbb {N}}_0^d}:\ \exists \,j\in {\mathbb {N}},\ \ \Vert \mathbf {c}\Vert _{\mathbf {M},j}<+\infty \},\\&\Lambda _{(\mathbf {M})}=\{\mathbf {c}=(c_\alpha )\in {\mathbb {C}}^{{\mathbb {N}}_0^d}:\ \forall \,j\in {\mathbb {N}},\ \Vert \mathbf {c}\Vert _{\mathbf {M},1/j}<+\infty \}. \end{aligned}$$

Now, for a weight matrix $\mathcal {M}$ as in (3.1), we denote

$$\begin{aligned}&\Lambda _{\{\mathcal {M}\}}:= \bigcup _{\lambda>0}\Lambda _{\{\mathbf {M}^{(\lambda )}\}} =\{\mathbf {c}=(c_\alpha )\in {\mathbb {C}}^{{\mathbb {N}}_0^d}:\ \exists \,\lambda ,h>0,\ \ \Vert \mathbf {c}\Vert _{\mathbf {M}^{(\lambda )},h}<+\infty \},\\&\Lambda _{(\mathcal {M})}:= \bigcap _{\lambda>0}\Lambda _{(\mathbf {M}^{(\lambda )})} =\{\mathbf {c}=(c_\alpha )\in {\mathbb {C}}^{{\mathbb {N}}_0^d}:\ \forall \, \lambda ,h>0,\ \ \Vert \mathbf {c}\Vert _{\mathbf {M}^{(\lambda )},h}<+\infty \}. \end{aligned}$$

Since $\mathbf {M}^{(\lambda )}\le \mathbf {M}^{(\kappa )}$ for $0<\lambda \le \kappa$ by assumption, then $\omega _{\mathbf {M}^{(\lambda )}}\ge \omega _{\mathbf {M}^{(\kappa )}}$. Moreover, $h\mapsto e^{\omega _{\mathbf {M}^{(\kappa )}}(\alpha ^{1/2}/h)}$ is decreasing for all $\kappa >0$, $\alpha \in {\mathbb {N}}_0^d$. It follows that we can write $\Lambda _{\{\mathcal {M}\}}$ ($\Lambda _{(\mathcal {M})}$) as inductive (projective) limit:

5.1 $$\begin{aligned}&\Lambda _{\{\mathcal {M}\}} =\{\mathbf {c}=(c_\alpha )\in {\mathbb {C}}^{{\mathbb {N}}_0^d}:\ \exists j\in {\mathbb {N}},\ \ \Vert \mathbf {c}\Vert _{\mathbf {M}^{(j)},j}<+\infty \}, \end{aligned}$$

5.2 $$\begin{aligned}&\Lambda _{(\mathcal {M})}=\{\mathbf {c}=(c_\alpha )\in {\mathbb {C}}^{{\mathbb {N}}_0^d}:\ \forall j\in {\mathbb {N}},\ \ \Vert \mathbf {c}\Vert _{\mathbf {M}^{(1/j)},1/j}<+\infty \}. \end{aligned}$$

Note that by Remark 1, it seems natural to require that $\lim _{|\alpha |\rightarrow \infty }(M_{\alpha })^{1/|\alpha |}=+\infty$ for the definition of $\Lambda _{\{\mathbf {M}\}}$ and $\Lambda _{(\mathbf {M})}$. In fact, otherwise $\omega _\mathbf {M}(t)=+\infty$ for all large $t\in {\mathbb {R}}^d$ and we get $\Lambda _{(\mathbf {M})}=\{0\}$ and $\Lambda _{\{\mathbf {M}\}}$ consisting of sequences having only finitely many values $\ne 0$.

However, in our next main result, by Remark 3 and assumption (3.2) ((3.3), respectively), we have the warranty of the finiteness of all associated weight functions under consideration.

### Theorem 1

*Let* $\mathcal {M}$ *be a weight matrix satisfying* (3.2) *and* (3.6). *Then the Hermite functions are an absolute Schauder basis in* $\mathcal {S}_{\{\mathcal {M}\}}$ *and*

$$\begin{aligned} T:\ \mathcal {S}_{\{\mathcal {M}\}}&\longrightarrow \Lambda _{\{\mathcal {M}\}}\\ f&\longmapsto (\xi _\gamma (f))_{\gamma \in {\mathbb {N}}_0^d}:= \left( \int _{{\mathbb {R}}^d}f(x)H_\gamma (x)dx\right) _{\gamma \in {\mathbb {N}}_0^d} \end{aligned}$$

*defines an isomorphism.*

*If* $\mathcal {M}$ *satisfies* (3.3) *and* (3.7), *then the Hermite functions are an absolute Schauder basis in* $\mathcal {S}_{(\mathcal {M})}$ *and the above defined operator* $T:\ \mathcal {S}_{(\mathcal {M})}\rightarrow \Lambda _{(\mathcal {M})}$ *is an isomorphism*.

### Proof

By Proposition 1 we can assume that $\mathcal {S}_{\{\mathcal {M}\}}$ and $\mathcal {S}_{(\mathcal {M})}$ are defined by $L^2$ norms. First, we consider the Roumieu case. If $f\in \mathcal {S}_{\{\mathcal {M}\}}$, there exist $\lambda ,C,C_1>0$ such that

$$\begin{aligned} \Vert f\Vert _{2,\mathbf {M}^{(\lambda )},C}=:C_1<+\infty . \end{aligned}$$

By (4.1) and Lemma 9, there exist $\kappa \ge \lambda$, $B,C,H>0$ such that for all $\gamma ,\alpha \in {\mathbb {N}}_0^d$, since $\Vert H_\gamma \Vert _2=1$ for all $\gamma \in {\mathbb {N}}_0^d$, we have

$$\begin{aligned} |\xi _\gamma (f)|^2\gamma ^\alpha&=|\langle f,H_\gamma \rangle |^2\gamma ^\alpha \le |\langle f,\sqrt{2^{|\alpha |}\gamma ^\alpha }H_\gamma \rangle |^2 =|\langle f,A_-^\alpha (H_{\gamma +\alpha })\rangle |^2\\ =&|\langle A_+^\alpha (f),H_{\gamma +\alpha }\rangle |^2\le \Vert A_+^\alpha (f)\Vert _2^2\Vert H_{\gamma +\alpha }\Vert _2^2\\ \le&C_1^2B^2e^d(9\sqrt{2}HC)^{2|\alpha |}(M^{(\kappa )}_\alpha )^2. \end{aligned}$$

Therefore, by definition of the associated weight function, and using the notation of (2.9), since $|(\gamma ^{1/2})^\alpha |=|\gamma _1^{\alpha _1/2}\cdots \gamma _d^{\alpha _d/2}| =(\gamma ^\alpha )^{1/2}$, we obtain

$$\begin{aligned} |\xi _\gamma (f)|e^{\omega _{\mathbf {M}^{(\kappa )}}(\gamma ^{1/2}/(9\sqrt{2}HC))} =&\sup _{\alpha \in {\mathbb {N}}_{0,\gamma }^d} \frac{|\xi _\gamma (f)|\left| \left( \frac{\gamma ^{1/2}}{9\sqrt{2}HC}\right) ^\alpha \right| }{M^{(\kappa )}_\alpha } \le C_1B e^{d/2}. \end{aligned}$$

Hence, $(\xi _\gamma (f))_\gamma \in \Lambda _{\{\mathcal {M}\}}$ and, more precisely, there exist $\kappa \ge \lambda$, $H,C>0$ and $B\ge 1$ such that

5.3 $$\begin{aligned} \Vert (\xi _\gamma (f))_\gamma \Vert _{\mathbf {M}^{(\kappa )},9\sqrt{2}HC}\le B e^{d/2} \Vert f\Vert _{2,\mathbf {M}^{(\lambda )},C}. \end{aligned}$$

This proves that *T* is continuous in the Roumieu case [32, Proposition 24.7].

On the other hand, given $\mathbf {c}=(c_\gamma )_{\gamma \in {\mathbb {N}}_0^d}\in \Lambda _{\{\mathcal {M}\}}$, let $\lambda ,C^*>0$ such that

$$\begin{aligned} \sup _{\gamma \in {\mathbb {N}}_0^d}|c_\gamma |e^{\omega _{\mathbf {M}^{(\lambda )}}(\gamma ^{1/2}/C^*)} =\Vert \mathbf {c}\Vert _{\mathbf {M}^{(\lambda )},C^*}=:C_1^*<+\infty . \end{aligned}$$

By Lemma 4, there exist $\kappa \ge \lambda$ and $B_1,B_2\ge 1$ such that

$$\begin{aligned} e^{-\omega _{\mathbf {M}^{(\lambda )}}(B_2t)+\omega _{\mathbf {M}^{(\kappa )}}(t)} \le B_1(1+|t|)^{-2(d+1)},\quad t\in {\mathbb {R}}^d. \end{aligned}$$

Then, by (3.2), there exist $\kappa '\ge \kappa$ and $B,C,H>0$ with $C\ge B_2C^*$, such that, by Lemma 10,

5.4 $$\begin{aligned} |c_\gamma |\cdot \Vert x^\alpha \partial ^\beta H_\gamma \Vert _2\le&|c_\gamma |(2HC)^{|\alpha +\beta |} M^{(\kappa ')}_{\alpha +\beta }Be^{\omega _{\mathbf {M}^{(\kappa )}}(\gamma ^{1/2}/C)}\nonumber \\ \le&C_1^*B(2HC)^{|\alpha +\beta |}M^{(\kappa ')}_{\alpha +\beta } e^{-\omega _{\mathbf {M}^{(\lambda )}}(\gamma ^{1/2}/C^*) +\omega _{\mathbf {M}^{(\kappa )}}(\gamma ^{1/2}/(B_2C^*))}\nonumber \\ \le&C_1^*BB_1(2HC)^{|\alpha +\beta |} M^{(\kappa ')}_{\alpha +\beta } \left( 1+\left| \frac{\gamma ^{1/2}}{B_2C^*}\right| \right) ^{-2(d+1)}. \end{aligned}$$

Since here $|\gamma ^{1/2}|$ denotes the Euclidean norm of the multi-index $\gamma ^{1/2}$, we have

5.5 $$\begin{aligned} |\gamma ^{1/2}|^{2(d+1)}=(\gamma _1+\ldots +\gamma _d)^{d+1} \ge |\gamma |^{d+1}. \end{aligned}$$

Hence,

$$\begin{aligned} \sum _{\gamma \in {\mathbb {N}}_0^d}|c_\gamma |\cdot \Vert x^\alpha \partial ^\beta H_\gamma \Vert _2\le&C_1^*BB_1(2HC)^{|\alpha +\beta |}M^{(\kappa ')}_{\alpha +\beta } \sum _{\gamma \in {\mathbb {N}}_0^d}\frac{1}{\left( 1+\left| \frac{\gamma ^{1/2}}{B_2C^*}\right| \right) ^{2(d+1)}}\\ \le&C_1^*BB_1(2HC)^{|\alpha +\beta |}M^{(\kappa ')}_{\alpha +\beta } \sum _{\gamma \in {\mathbb {N}}_0^d}\frac{1}{1+\left| \frac{\gamma ^{1/2}}{B_2C^*}\right| ^{2(d+1)}}\\ =&C_1^*BB_1(2HC)^{|\alpha +\beta |}M^{(\kappa ')}_{\alpha +\beta } \sum _{\gamma \in {\mathbb {N}}_0^d}\frac{(B_2C^*)^{2(d+1)}}{(B_2C^*)^{2(d+1)}+|\gamma |^{d+1}}. \end{aligned}$$

Hence,

5.6 $$\begin{aligned} \left\| \sum \nolimits _{\gamma \in {\mathbb {N}}_0^d}c_\gamma H_\gamma \right\| _{2,\mathbf {M}^{(\kappa ')},2HC} \le C_1^*BB_1\tilde{C} =BB_1\tilde{C}\Vert \mathbf {c}\Vert _{\mathbf {M}^{(\lambda )},C^*}, \end{aligned}$$

for $\tilde{C}=\sum _{\gamma \in {\mathbb {N}}_0^d} (B_2C^*)^{2(d+1)}/((B_2C^*)^{2(d+1)}+|\gamma |^{d+1})<+\infty$. This shows that $T^{-1}$ is continuous and, moreover, that $(H_\gamma )_\gamma$ is an absolute Schauder basis in $\mathcal {S}_{\{\mathcal {M}\}}$.

Let now $f\in \mathcal {S}_{(\mathcal {M})}$ and $\lambda ,C>0$ be given. We consider $0<\kappa \le \lambda$, $H,B>0$ as in (3.3) (with $\kappa$ and *H* depending only on $\lambda$) and we set

$$\begin{aligned} C_1:=\Vert f\Vert _{2,\mathbf {M}^{(\kappa )},C}<+\infty . \end{aligned}$$

By Lemma 8, we have

$$\begin{aligned} \Vert A_+^\alpha f\Vert _2\le C_1B e^{d/2}(9\sqrt{2}HC)^{|\alpha |}M^{(\lambda )}_\alpha , \quad \alpha \in {\mathbb {N}}_0^d. \end{aligned}$$

Hence, proceeding as in the Roumieu case, we deduce that, for all $\lambda ,C>0$, there exist $0<\kappa \le \lambda$ and $B,H>0$ such that

5.7 $$\begin{aligned} \Vert (\xi _\gamma (f))_\gamma \Vert _{\mathbf {M}^{(\lambda )},9\sqrt{2}HC}\le B e^{d/2} \Vert f\Vert _{2,\mathbf {M}^{(\kappa )},C}. \end{aligned}$$

This shows that *T* is continuous in the Beurling case.

Now, if $\mathbf {c}=(c_\gamma )_{\gamma \in {\mathbb {N}}_0^d}\in \Lambda _{(\mathcal {M})}$, then by (3.3) and Lemma 10, for all $\lambda ,C>0$ there exist $0<\kappa \le \lambda$, and $H,B>0$ (with $\kappa$ and *H* depending only on $\lambda$) such that

$$\begin{aligned} \Vert x^\alpha \partial ^\beta H_\gamma \Vert _2\le (2HC)^{|\alpha +\beta |} M^{(\lambda )}_{\alpha +\beta }Be^{\omega _{\mathbf {M}^{(\kappa )}}(\gamma ^{1/2}/C)}. \end{aligned}$$

By Lemma 4, there exist $0<\kappa '\le \kappa$ and $B_1,B_2\ge 1$ such that

$$\begin{aligned} e^{-\omega _{\mathbf {M}^{(\kappa ')}}(B_2t)+\omega _{\mathbf {M}^{(\kappa )}}(t)} \le B_1(1+|t|)^{-2(d+1)}, \ \ t\in {\mathbb {R}}^d. \end{aligned}$$

Since $\mathbf {c}\in \Lambda _{(\mathcal {M})}$, we have

$$\begin{aligned} \sup _{\gamma \in {\mathbb {N}}_0^d}|c_\gamma |e^{\omega _{\mathbf {M}^{(\kappa ')}}(B_2\gamma ^{1/2}/C)} =\Vert \mathbf {c}\Vert _{\mathbf {M}^{(\kappa ')},C/B_2}=:C_1<+\infty . \end{aligned}$$

Therefore, arguing as in the Roumieu case,

$$\begin{aligned} \sum _{\gamma \in {\mathbb {N}}_0^d}|c_\gamma |\cdot \Vert x^\alpha \partial ^\beta H_\gamma \Vert _2\le&C_1B(2HC)^{|\alpha +\beta |}M^{(\lambda )}_{\alpha +\beta }\\&\cdot \sum _{\gamma \in {\mathbb {N}}_0^d} e^{-\omega _{\mathbf {M}^{(\kappa ')}}(B_2\gamma ^{1/2}/C)+\omega _{\mathbf {M}^{(\kappa )}}(\gamma ^{1/2}/C)}\\ \le&C_1BB_1(2HC)^{|\alpha +\beta |}M^{(\lambda )}_{\alpha +\beta } \sum _{\gamma \in {\mathbb {N}}_0^d}\frac{1}{(1+|\gamma ^{1/2}/C|)^{2(d+1)}}\\ \le&\tilde{B}C_1(2HC)^{|\alpha +\beta |}M^{(\lambda )}_{\alpha +\beta }, \end{aligned}$$

for $\tilde{B}=BB_1\sum _{\gamma \in {\mathbb {N}}_0^d}C^{2(d+1)}/(C^{2(d+1)}+|\gamma |^{d+1}) <+\infty$. For all $\lambda ,h>0$, there exist then $\kappa '\le \lambda$ and $\tilde{h}=h/(2HB_2)>0$ such that

5.8 $$\begin{aligned} \left\| \sum \nolimits _{\gamma \in {\mathbb {N}}_0^d}c_\gamma H_\gamma \right\| _{2,\mathbf {M}^{(\lambda )},h} \le \tilde{B}\Vert \mathbf {c}\Vert _{\mathbf {M}^{(\kappa ')},{\tilde{h}}}. \end{aligned}$$

This shows that $T^{-1}$ is continuous on $\mathcal {S}_{(\mathcal {M})}$ and that $(H_\gamma )_\gamma$ is an absolute Schauder basis in $\mathcal {S}_{(\mathcal {M})}$, which finishes the proof. $\square$

As in [28, Corollary 3.6], we also have that the Fourier transform is well adapted to our spaces and it is an isomorphism:

### Corollary 1

*Let* $\mathcal {M}$ *be a weight matrix satisfying* (3.2) *and* (3.6) ((3.3) *and* (3.7)). *Then the Fourier transform is an isomorphism in* $\mathcal {S}_{\{\mathcal {M}\}}$ ($\mathcal {S}_{(\mathcal {M})}$).

Now, we prove that the spaces of sequences are nuclear.

### Theorem 2

*Let* $\mathcal {M}=(M^{(\lambda )}_\alpha )_{\lambda >0,\alpha \in {\mathbb {N}}_0^d}$ *be a weight matrix satisfying* (3.7). *Then* $\Lambda _{(\mathcal {M})}$ *is nuclear*.

### Proof

By (5.2) and [32, Prop. 28.16] (see also [10, Theorem 3.1] for a self-contained proof in the case of countable lattices), the sequence space $\Lambda _{(\mathcal {M})}$ is nuclear if and only if

5.9 $$\begin{aligned} \forall j\in {\mathbb {N}}\,\exists \ell \in {\mathbb {N}},\ell >j: \ \ \ \sum _{\gamma \in {\mathbb {N}}^d_0} e^{\omega _{\mathbf {M}^{(1/j)}}(j\gamma ^{1/2})-\omega _{\mathbf {M}^{(1/\ell )}}(\ell \gamma ^{1/2})} <+\infty . \end{aligned}$$

Moreover, by Lemma 5, condition (3.14) is satisfied. We can thus proceed as in the proof of Theorem 1 of [9] to prove that (3.14) implies that the series in (5.9) converges, and hence $\Lambda _{(\mathcal {M})}$ is nuclear. To this aim, we fix an index $\lambda >0$ and $N\in {\mathbb {N}}$ with $N>2d$ and remark that if the inequality (3.14) holds for $\lambda =1/j$ and $\kappa \le \lambda$, then it holds also if, instead of $\kappa$, we put $\kappa '=1/h$ with $h\in {\mathbb {N}}$, $h>[\frac{1}{\kappa }]+1$, since $\mathbf {M}^{(\kappa ')}\le \mathbf {M}^{(\kappa )}$ for $\kappa '\le \kappa$ and hence $\omega _{\mathbf {M}^{(\kappa )}}\le \omega _{\mathbf {M}^{(\kappa ')}}$. Then for $\ell \ge Ah$ (so that $\ell \ge Aj$ and $\ell \ge h>j$ and note that the constant *A* is also depending on the chosen *N*):

$$\begin{aligned}&\sum _{\gamma \in {\mathbb {N}}^d_0}e^{\omega _{\mathbf {M}^{(1/j)}}(j\gamma ^{1/2})- \omega _{\mathbf {M}^{(1/\ell )}}(\ell \gamma ^{1/2})} \le \sum _{\gamma \in {\mathbb {N}}^d_0\backslash \{0\}}e^{\omega _{\mathbf {M}^{(1/j)}} (j\gamma ^{1/2})-\omega _{\mathbf {M}^{(1/h)}}(A j\gamma ^{1/2})}+1\\&\ \ \ \ \ \ \ \ \ \ \ \ \le \sum _{\gamma \in {\mathbb {N}}^d_0\backslash \{0\}}e^{-N\log |j\gamma ^{1/2}|+B} +1=e^Bj^{-N}\sum _{\gamma \in {\mathbb {N}}^d_0\backslash \{0\}} \frac{1}{\vert \gamma \vert ^{N/2}}+1<+\infty , \end{aligned}$$

by our choice of $N>2d$. $\square$

Concerning the Roumieu case, we have the following result.

### Theorem 3

*Let* $\mathcal {M}=(M^{(\lambda )}_\alpha )_{\lambda >0,\alpha \in {\mathbb {N}}_0^d}$ *be a weight matrix satisfying* (3.6). *Then* $\Lambda _{\{\mathcal {M}\}}$ *is nuclear*.

### Proof

For

$$\begin{aligned} a_{\alpha ,j}:= e^{-\omega _{\mathbf {M}^{(j)}}(\alpha ^{1/2}/j)}, \end{aligned}$$

we consider the matrices

$$\begin{aligned} A:=\left( a_{\alpha ,j}\right) _{\alpha \in {\mathbb {N}}_0^d,\ j\in {\mathbb {N}}},\qquad V:=\left( v_{\alpha ,j}\right) _{\alpha \in {\mathbb {N}}_0^d,\ j\in {\mathbb {N}}}\ \text {with}\ v_{\alpha ,j}=a_{\alpha ,j}^{-1}. \end{aligned}$$

We observe that *A* is a Köthe matrix since its entries are strictly positive and $a_{\alpha ,j}\le a_{\alpha ,j+1}$ for every $j\in {\mathbb {N}}$. We consider now the space

$$\begin{aligned} \lambda _{(\mathcal {M})}:=\{\mathbf{c}=(c_\alpha )\in {\mathbb {C}}^{{\mathbb {N}}_0^d}:\ \forall j\in {\mathbb {N}},\ \sum _{\alpha \in {\mathbb {N}}_0^d} |c_\alpha | a_{\alpha ,j}<\infty \}. \end{aligned}$$

Since ${\mathbb {N}}_0^d = \cup _{m\in {\mathbb {N}}}I_m$ with $I_m=\{\alpha \in {\mathbb {N}}_0^d : |\alpha |\le m\}$ and $v_{\alpha ,j}>0$ for every $\alpha$ and *j*, we have that the matrix *V* satisfies the *condition (D)* of [5] (see also [4]). From [4, Theorem 18(1)], we have that $\lambda _{(\mathcal {M})}$ is distinguished, and then, from [4, Corollary 8*(f)*] and (5.1) we get

$$\begin{aligned} \left( \lambda _{(\mathcal {M})}\right) _b' = \Lambda _{\{\mathcal {M}\}}. \end{aligned}$$

Since a Fréchet space is nuclear if and only if its dual is nuclear [34, pg. 78], it is enough to prove that $\lambda _{(\mathcal {M})}$ is nuclear; from [10, Theorem 3.1] this is true if and only if

5.10 $$\begin{aligned} \forall k\in {\mathbb {N}}\,\exists m\in {\mathbb {N}},m>k:\ \ \sum _{\gamma \in {\mathbb {N}}^d_0} e^{\omega _{\mathbf {M}^{(m)}}(\gamma ^{1/2}/m)-\omega _{\mathbf {M}^{(k)}}(\gamma ^{1/2}/k)} <+\infty . \end{aligned}$$

By Lemma 5, we can now use (3.15) with $\lambda =k$ and with a fixed $N>2d$; since $\omega _{\mathbf {M}^{(m)}}(t)\le \omega _{\mathbf {M}^{(\kappa )}}(t)$ for every $m\ge \kappa$, we can replace in (3.15) $\kappa$ by $m=\max \{\kappa ,Ak\}$, obtaining that for every $k\in {\mathbb {N}}$ there exists $m\ge k$ such that

$$\begin{aligned} \omega _{\mathbf {M}^{(m)}}\left( \frac{\gamma ^{1/2}}{m}\right) +N\log \left| \frac{\gamma ^{1/2}}{m}\right| \le \omega _{\mathbf {M}^{(k)}}\left( A\frac{\gamma ^{1/2}}{m}\right) +B, \end{aligned}$$

for every $\gamma \ne 0$. Since $A\le m/k$ we obtain

$$\begin{aligned} e^{\omega _{\mathbf {M}^{(m)}}(\gamma ^{1/2}/m)-\omega _{\mathbf {M}^{(k)}}(\gamma ^{1/2}/k)}\le e^B m^N\frac{1}{|\gamma ^{1/2}|^N}\le e^B m^N\frac{1}{|\gamma |^{N/2}}, \end{aligned}$$

for $\gamma \ne 0$; since $N>2d$ we have that (5.10) holds, and estimating as in Theorem 2, we get the conclusion. $\square$

### Corollary 2

*If* $\mathcal {M}=(M^{(\lambda )}_\alpha )_{\lambda >0,\alpha \in {\mathbb {N}}_0^d}$ *is a weight matrix satisfying* (3.3) *and* (3.7), *then the space* $\mathcal {S}_{(\mathcal {M})}$ *is nuclear. If* $\mathcal {M}$ *satisfies* (3.2) *and* (3.6), *then the space* $\mathcal {S}_{\{\mathcal {M}\}}$ *is nuclear*.

### Proof

The Beurling case follows from Theorems 1 and 2, and the Roumieu case follows from Theorems 1 and 3.$\square$

### Proposition 4

*Let* $\mathcal {M}=(M^{(\lambda )}_p)_{\lambda >0,p\in {\mathbb {N}}_0}$ *be a weight matrix* (*with* $d=1$), *such that each sequence* $\mathbf {M}^{(\lambda )}$ *satisfies* (2.2) *and* $\lim _{p\rightarrow \infty }(M_p)^{1/p}=+\infty$. *Assume, moreover, that*

$$\begin{aligned} \mu ^{(\lambda )}_p:=\frac{M^{(\lambda )}_p}{M^{(\lambda )}_{p-1}},\qquad p\in {\mathbb {N}}, \end{aligned}$$

*satisfies* $\mu ^{(\lambda )}\le \mu ^{(\kappa )}$ *for all* $0<\lambda \le \kappa$ *and* $\mu ^{(\lambda )}_0=1$ *for all* $\lambda >0$. *Then the following conditions are equivalent*:

- $$\forall j\in {\mathbb {N}}\ \exists \,\ell \in {\mathbb {N}},\ell >j:\ \ \sum _{k=1}^{+\infty } e^{\omega _{\mathbf {M}^{(1/j)}}(jk^{1/2})-\omega _{\mathbf {M}^{(1/\ell )}}(\ell k^{1/2})}<+\infty$$;
- $$\forall \;\lambda >0\ \exists \;0<\kappa <\lambda , A\ge 1\ \forall \,p\in {\mathbb {N}}: \ \ M^{(\kappa )}_{p+1}\le A^{p+1}M^{(\lambda )}_p.$$

### Proof

If condition (*b*) is satisfied, then (3.7) is satisfied and hence also condition (*a*), as we already saw in the proof of Theorem 2.

Let us now assume condition (a) and prove (b). To this aim let us first remark that

5.11 $$\begin{aligned} k\longmapsto \omega _{\mathbf {M}^{(1/j)}}(jk^{1/2})-\omega _{\mathbf {M}^{(1/\ell )}}(\ell k^{1/2}) \end{aligned}$$

is decreasing. Indeed,

$$\begin{aligned} \omega _{\mathbf {M}^{(1/\ell )}}(\ell k^{1/2})-\omega _{\mathbf {M}^{(1/j)}}(j k^{1/2})= & {} \left( \omega _{\mathbf {M}^{(1/\ell )}}(\ell k^{1/2})- \omega _{\mathbf {M}^{(1/\ell )}}(j k^{1/2})\right) \\&+\left( \omega _{\mathbf {M}^{(1/\ell )}}(j k^{1/2})- \omega _{\mathbf {M}^{(1/j)}}(j k^{1/2})\right) \\=: & {} \omega _1+\omega _2. \end{aligned}$$

The first difference $\omega _1=\omega _{\mathbf {M}^{(1/\ell )}}(\ell k^{1/2})-\omega _{\mathbf {M}^{(1/\ell )}}(j k^{1/2})$ is increasing since by definition $t\mapsto \omega _{\mathbf {M}^{(1/\ell )}}(e^t)$ is convex (see the proof of Theorem 1 in [9] for the implication that the convexity implies that $\omega _1$ is increasing).

To prove that also the second difference $\omega _2$ is increasing, we set

$$\begin{aligned} \Sigma _{\mathbf {M}^{(\lambda )}}(t):=\#\{p\in {\mathbb {N}}:\ \mu ^{(\lambda )}_p\le t\} \end{aligned}$$

and remark that, by (2.2) (see [26, formula (3.11)]),

$$\begin{aligned} \omega _{\mathbf {M}^{(\lambda )}}(t)=\int _0^t \frac{\Sigma _{\mathbf {M}^{(\lambda )}}(s)}{s}ds. \end{aligned}$$

Then

$$\begin{aligned} \omega _{\mathbf {M}^{(1/\ell )}}(t)-\omega _{\mathbf {M}^{(1/j)}}(t) =\int _0^t\frac{\Sigma _{\mathbf {M}^{(1/\ell )}}(s)-\Sigma _{\mathbf {M}^{(1/j)}}(s)}{s}ds \end{aligned}$$

is an increasing function of *t* since

$$\begin{aligned} \Sigma _{\mathbf {M}^{(1/\ell )}}(s)\ge \Sigma _{\mathbf {M}^{(1/j)}}(s),\qquad \ell >j, \end{aligned}$$

by the assumption $\mu ^{(1/\ell )}_p\le \mu ^{(1/j)}_p$ for $\ell >j$.

Therefore, $\omega _1$ and $\omega _2$ are increasing and we have thus proved that (5.11) is decreasing. This condition together with assumption (*a*) implies that

$$\begin{aligned} \lim _{k\rightarrow +\infty }k e^{\omega _{\mathbf {M}^{(1/j)}}(jk^{1/2})-\omega _{\mathbf {M}^{(1/\ell )}}(\ell k^{1/2})}=0. \end{aligned}$$

There exists then $A\ge 1$ such that

$$\begin{aligned} \sup _{k\in {\mathbb {N}}}ke^{\omega _{\mathbf {M}^{(1/j)}}(jk^{1/2}) -\omega _{\mathbf {M}^{(1/\ell )}}(\ell k^{1/2})}\le A \end{aligned}$$

and hence, for all $k\in {\mathbb {N}}$,

$$\begin{aligned} \omega _{\mathbf {M}^{(1/j)}}(jk^{1/2})-\omega _{\mathbf {M}^{(1/\ell )}}(\ell k^{1/2}) \le -\log k+\log A\le -\log (jk^{1/2})+\log (jA). \end{aligned}$$

Choosing, for every $t\ge 1$, the smallest $k\in {\mathbb {N}}$ such that $jk^{1/2}\in [t,(j+1)t]$, we finally have

5.12 $$\begin{aligned} \omega _{\mathbf {M}^{(1/j)}}(t)+\log t\le&\omega _{\mathbf {M}^{(1/j)}}(jk^{1/2})+\log (jk^{1/2})\nonumber \\ \le&\omega _{\mathbf {M}^{(1/\ell )}}(\ell k^{1/2})+\log (jA)\nonumber \\ \le&\omega _{\mathbf {M}^{(1/\ell )}}\left( \frac{\ell }{j}(j+1) t\right) +\log (jA). \end{aligned}$$

Since (5.12) is trivial for $0<t\le 1$, we have proved that condition (*ii*) of Lemma 2 is satisfied for $\mathbf {N}=\mathbf {M}^{(1/j)}$ and $\mathbf {M}=\mathbf {M}^{(1/\ell )}$ and hence, from (*i*) of Lemma 2, there exists ${\tilde{A}}\ge 1$ such that

$$\begin{aligned} M^{(1/\ell )}_{p+1}\le \tilde{A}^{p+1}M^{(1/j)}_p,\qquad \forall p\in {\mathbb {N}}_0. \end{aligned}$$

Then, for all $\lambda >0$, choosing $j\in {\mathbb {N}}$ so that $\frac{1}{j}\le \lambda$, there exists $\kappa =\frac{1}{\ell }<\frac{1}{j}\le \lambda$ such that condition (b) holds.$\square$

Proposition 4 yields now the following result.

### Theorem 4

*Let* $\mathcal {M}=(M^{(\lambda )}_p)_{\lambda >0,p\in {\mathbb {N}}_0}$ *be a weight matrix as in Proposition* 4. *Then the space* $\Lambda _{(\mathcal {M})}$ *is nuclear if and only if condition* (3.7) *is satisfied*.

### Proof

It follows from Theorem 2 and, in particular, (5.9).$\square$

### Theorem 5

*Let* $\mathcal {M}=(M^{(\lambda )}_p)_{\lambda >0,p\in {\mathbb {N}}_0}$ *be a weight matrix as in Proposition* 4. *Then the space* $\Lambda _{\{\mathcal {M}\}}$ *is nuclear if and only if condition* (3.6) *is satisfied*.

### Proof

By the proof of Theorem 3 we have that $\Lambda _{\{\mathcal {M}\}}$ is nuclear if and only if (5.10) is satisfied, and this is equivalent to (3.6) since, analogously as in Proposition 4, the following two conditions are equivalent:

$$(a)'$$
$$\forall \, j\in {\mathbb {N}}\ \exists \,\ell \in {\mathbb {N}},\ell >j:\ \ \sum _{k=1}^{+\infty } e^{\omega _{\mathbf {M}^{(\ell )}}(k^{1/2}/\ell )-\omega _{\mathbf {M}^{(j)}}(k^{1/2}/j)}<+\infty$$,
$$(b)'$$
$$\forall \,\lambda>0\ \exists \,\kappa >\lambda , A\ge 1\ \forall \,p\in {\mathbb {N}}:\ \ M^{(\lambda )}_{p+1}\le A^{p+1}M^{(\kappa )}_p$$.

Indeed, $(b)'$ implies (3.6) and hence $(a)'$, i.e. (5.10), in the one-dimensional case, by the proof of Theorem 3.

Conversely, if (a)′ holds then for every fixed $j\in {\mathbb {N}}$, and $\ell >j$ as in (a)′, there exists $A>\ell$ such that

$$\begin{aligned} \sup _{k\in {\mathbb {N}}}ke^{\omega _{\mathbf {M}^{(\ell )}}(k^{1/2}/\ell )- \omega _{\mathbf {M}^{(j)}}(k^{1/2}/j)}\le A \end{aligned}$$

since

$$\begin{aligned} k\longmapsto \omega _{\mathbf {M}^{(\ell )}}(k^{1/2}/\ell )-\omega _{\mathbf {M}^{(j)}}(k^{1/2}/j) \end{aligned}$$

is decreasing, similarly as in the proof of Proposition 4. Then, for all $k\in {\mathbb {N}}$,

$$\begin{aligned} \omega _{\mathbf {M}^{(\ell )}}(k^{1/2}/\ell )-\omega _{\mathbf {M}^{(j)}}(k^{1/2}/j) \le -\log k+\log A\le -\log (k^{1/2}/\ell )+\log (A/\ell ). \end{aligned}$$

If $t\ge 1$ we can choose a smallest $k\in {\mathbb {N}}$ such that $k^{1/2}/\ell \in [t,(1+\frac{1}{\ell })t]$ and obtain that

5.13 $$\begin{aligned} \omega _{\mathbf {M}^{(\ell )}}(t)+\log t\le&\omega _{\mathbf {M}^{(\ell )}} (k^{1/2}/\ell )+\log (k^{1/2}/\ell )\nonumber \\ \le&\omega _{\mathbf {M}^{(j)}}(k^{1/2}/j)+\log (A/\ell )\nonumber \\ \le&\omega _{\mathbf {M}^{(j)}}\left( \frac{\ell }{j}\left( 1+\frac{1}{\ell }\right) t\right) +\log (A/\ell ). \end{aligned}$$

Since (5.13) is trivial for $0<t\le 1$, we have that

$$\begin{aligned} \omega _{\mathbf {M}^{(\ell )}}(t)+\log t\le \omega _{\mathbf {M}^{(j)}}(At)+B,\qquad \forall t>0, \end{aligned}$$

for $A=\frac{\ell }{j}\left( 1+\frac{1}{\ell }\right) \ge 1$ and $B=\log (A/\ell )>0$. By Lemma 2 with $\mathbf {M}=\mathbf {M}^{(j)}$ and $\mathbf {N}=\mathbf {M}^{(\ell )}$, for every $\lambda >0$ we can choose $j\in {\mathbb {N}}$, $j\ge \lambda$ so that $(b)'$ is satisfied for $\kappa =\ell >j\ge \lambda$. $\square$

## Rapidly decreasing ultradifferentiable functions

We shall now consider weight functions $\omega$ defined as below:

### Definition 1

A *weight function* is a continuous increasing function $\omega \!:[0,+\infty )\rightarrow [0,+\infty )$ such that

$$(\alpha )$$
$$\exists L\ge 1\ \forall t\ge 0:\ \omega (2t)\le L(\omega (t)+1)$$;
$$(\beta )$$
$$\omega (t)=O(t^2)$$ as $$t\rightarrow +\infty$$;
$$(\gamma )$$
$$\log t=o(\omega (t))$$ as $$t\rightarrow +\infty$$;
$$(\delta )$$
$\varphi _\omega (t):=\omega (e^t)$ is convex on $[0,+\infty )$.

Then we define $\omega (t):=\omega (|t|)$ if $t\in {\mathbb {R}}^d$.

It is not restrictive to assume $\omega |_{[0,1]}\equiv 0$. As usual, we define the Young conjugate $\varphi ^*_\omega$ of $\varphi _\omega$ by

$$\begin{aligned} \varphi ^*_\omega (s):=\sup _{t\ge 0}\{ts-\varphi _\omega (t)\}, \end{aligned}$$

which is an increasing convex function such that $$\varphi ^{**}_\omega =\varphi _\omega$$ and $$\varphi ^*(s)/s$$ is increasing [12, 24]. We remark that condition $$(\beta )$$ and the stronger condition $$\omega (t)=o(t^2)$$ as *t* tends to infinity are needed in the Roumieu and Beurling cases for Corollary 5 and Theorem 6. On the other hand, condition $$(\gamma )$$ guarantees that $$\varphi ^*_\omega$$ is finite, so that, from the properties of $$\varphi ^*_\omega$$ (see [12] or [8, Lemma A.1]) we easily obtain (cf. [37]):

### Lemma 11

*Let* $\omega :\ [0,+\infty )\rightarrow [0,+\infty )$ *be a weight function as in Definition* 1, *and set*

6.1 $$\begin{aligned} W^{(\lambda )}_\alpha :=e^{\frac{1}{\lambda }\varphi ^*_\omega (\lambda |\alpha |)}, \qquad \lambda >0,\alpha \in {\mathbb {N}}_0^d. \end{aligned}$$

*Then* $W^{(\lambda )}_\alpha \in {\mathbb {R}}$ *and the weight matrix*

6.2 $$\begin{aligned} \mathcal {M}_\omega :=(\mathbf {W}^{(\lambda )})_{\lambda>0}=(W^{(\lambda )}_\alpha )_{\lambda >0,\, \alpha \in {\mathbb {N}}_0^d} \end{aligned}$$

*satisfies the following properties*:

(i) $$W^{(\lambda )}_0=1,\quad \lambda >0$$;
(ii) $(W^{(\lambda )}_\alpha )^2\le W^{(\lambda )}_{\alpha -e_i}W^{(\lambda )}_{\alpha +e_i},\quad \lambda >0,\alpha \in {\mathbb {N}}^d_0$ with $\alpha _i\ne 0$, *and* $i=1,\dots ,d$;
(iii) $$\mathbf {W}^{(\kappa )}\le \mathbf {W}^{(\lambda )},\quad 0<\kappa \le \lambda$$;
(iv) $$W^{(\lambda )}_{\alpha +\beta }\le W^{(2\lambda )}_\alpha W^{(2\lambda )}_\beta ,\quad \lambda >0,\alpha ,\beta \in {\mathbb {N}}_0^d$$;
(v) $$\forall h>0\ \exists A\ge 1\ \forall \lambda >0\ \exists D\ge 1\ \forall \alpha \in {\mathbb {N}}_0^d:\ \ \ h^{|\alpha |}W^{(\lambda )}_\alpha \le DW^{(A\lambda )}_\alpha ;$$
(vi) *Both conditions* (3.6) *and* (3.7) *are valid;*
(vii) *Conditions* (3.4) *and* (3.5) *are satisfied for* $\kappa =\lambda$ *and* $A=1$.

### Proof

Let us first remark that condition $(\gamma )$ of Definition 1 ensures that $W^{(\lambda )}_\alpha \in {\mathbb {R}}$ for all $\lambda >0$ and $\alpha \in {\mathbb {N}}_0^d$. Condition (i) is trivial since $\varphi ^*_\omega (0)=0$. Condition (ii) follows from the convexity of $\varphi ^*_\omega$:

$$\begin{aligned} e^{\frac{2}{\lambda }\varphi ^*_\omega (\lambda |\alpha |)}= e^{\frac{2}{\lambda }\varphi ^*_\omega \left( \frac{\lambda (|\alpha |-1)+ \lambda (|\alpha |+1)}{2}\right) } \le e^{\frac{1}{\lambda }\varphi ^*_\omega (\lambda |\alpha -e_i|)}e^{\frac{1}{\lambda } \varphi ^*_\omega (\lambda |\alpha +e_i|)}. \end{aligned}$$

The monotonicity property (iii) is clear since $\varphi ^*_\omega (s)/s$ is increasing. Properties (iv), (v) and (vii) follow from [8, Lemma A.1]. Indeed, from [8, Lemma A.1(ix)]

$$\begin{aligned} e^{\frac{1}{\lambda }\varphi ^*_\omega (\lambda |\alpha +\beta |)}\le e^{\frac{1}{2\lambda }\varphi ^*_\omega (2\lambda |\alpha |) +\frac{1}{2\lambda }\varphi ^*_\omega (2\lambda |\beta |)}. \end{aligned}$$

From [8, Lemma A.1(iv)] with $A=L^2+L$ and $B=L^2$, where *L* is the constant of condition $(\alpha )$ of Definition 1,

$$\begin{aligned} h^{|\alpha |}e^{\frac{1}{\lambda }\varphi ^*_\omega (\lambda |\alpha |)} \le \Lambda _{h,\lambda }e^{\frac{1}{\lambda '}\varphi ^*_\omega (\lambda ' |\alpha |)} \end{aligned}$$

for all $\lambda '\ge \lambda B^{[\log h+1]}$ and $\Lambda _{h,\lambda }:= e^{\frac{1}{\lambda }\left( 1+\frac{1}{L}\right) [\log h+1]}$. From [8, Lemma A.1(ii)]

$$\begin{aligned} e^{\frac{1}{\lambda }\varphi ^*_\omega (\lambda |\alpha |)+\frac{1}{\lambda } \varphi ^*_\omega (\lambda |\beta |)}\le e^{\frac{1}{\lambda }\varphi ^*_\omega (\lambda |\alpha +\beta |)}. \end{aligned}$$

Finally, (vi) is an immediate consequence of (iv).$\square$

Let us now define the spaces of rapidly decreasing $\omega$-ultradifferentiable functions, in the Roumieu case

$$\begin{aligned} \mathcal {S}_{\{\omega \}}({\mathbb {R}}^d):=&\big \{f\in C^\infty ({\mathbb {R}}^d):\ \exists \lambda>0,C>0\ \text{ s.t. }\\&\sup _{\alpha ,\beta \in {\mathbb {N}}_0^d}\Vert x^\alpha \partial ^\beta f\Vert _\infty e^{-\frac{1}{\lambda }\varphi ^*_\omega (\lambda |\alpha +\beta |)}\le C\big \}\\ =&\big \{f\in C^\infty ({\mathbb {R}}^d):\ \exists \lambda>0,C>0\ \text{ s.t. }\\& \Vert f\Vert _{\infty ,\mathbf {W}^{(\lambda )}}:= \sup _{\alpha ,\beta \in {\mathbb {N}}_0^d}\frac{\Vert x^\alpha \partial ^\beta f\Vert _\infty }{W^{(\lambda )}_{\alpha +\beta }}\le C\big \}, \end{aligned}$$

and in the Beurling case

$$\begin{aligned} \mathcal {S}_{(\omega )}({\mathbb {R}}^d):=&\big \{f\in C^\infty ({\mathbb {R}}^d):\ \forall \lambda>0\,\exists C_\lambda >0:\ \Vert f\Vert _{\infty ,\mathbf {W}^{(\lambda )}}\le C_\lambda \big \}. \end{aligned}$$

From Lemma 11(iv) and (vii) (see also [6, Thm. 4.8]):

$$\begin{aligned} \mathcal {S}_{\{\omega \}}({\mathbb {R}}^d)=\big \{f\in C^\infty ({\mathbb {R}}^d):\ \exists \lambda>0,C>0:\ \sup _{\alpha ,\beta \in {\mathbb {N}}_0^d} \frac{\Vert x^\alpha \partial ^\beta f\Vert _\infty }{W^{(\lambda )}_{\alpha } W^{(\lambda )}_{\beta }}\le C\big \} \end{aligned}$$

and

$$\begin{aligned} \mathcal {S}_{(\omega )}({\mathbb {R}}^d)=\big \{f\in C^\infty ({\mathbb {R}}^d):\ \forall \lambda>0\,\exists C_\lambda >0:\ \sup _{\alpha ,\beta \in {\mathbb {N}}_0^d}\frac{\Vert x^\alpha \partial ^\beta f\Vert _\infty }{W^{(\lambda )}_{\alpha } W^{(\lambda )}_{\beta }}\le C_\lambda \big \}. \end{aligned}$$

We refer to [6, 8, 22] for more equivalent seminorms on $\mathcal {S}_{(\omega )}({\mathbb {R}}^d)$, if $\omega (t)=o(t^2)$.

We can also insert $h^{|\alpha +\beta |}$ at the denominator (for some $h>0$ in the Roumieu case and for all $h>0$ in the Beurling case) by Lemma 11(v). In particular, we have the following

### Proposition 5

*Let* $\omega$ *be a weight function and* $\mathcal {M}_\omega$ *the weight matrix defined in* (6.1), (6.2). *We have* $\mathcal {S}_{\{\mathcal {M}_\omega \}}({\mathbb {R}}^d)=\mathcal {S}_{\{\omega \}}({\mathbb {R}}^d)$ *and* $\mathcal {S}_{(\mathcal {M}_\omega )}({\mathbb {R}}^d)=\mathcal {S}_{(\omega )}({\mathbb {R}}^d)$ *and the equalities are also topological*.

### Remark 4

We observe that for the weight function $\omega (t)=\log ^s(1+t)$, for some $s>1$, we have that $\mathcal {S}_{(\omega )}({\mathbb {R}})$ never equals $\mathcal {S}_{(M_p)}({\mathbb {R}})$ for any sequence $(M_p)_{p\in {\mathbb {N}}_0}$. Hence, $\mathcal {S}_{(\omega )}({\mathbb {R}})$ cannot be defined with sequences as in [28] when $(M_p)$ satisfies (*M*0), (*M*1) and $(M2)'$ (see [11] for the definition of (*M*0); (*M*1) and $(M2)'$ are recalled in (2.2) and (2.4)).

Indeed, by [11, Example 20], $\mathcal {E}_{(\omega )}({\mathbb {R}})\ne \mathcal {E}_{(M_p)}({\mathbb {R}})$ for any sequence $(M_p)$ as considered just above, where $\mathcal {E}_{(\omega )}({\mathbb {R}})$ and $\mathcal {E}_{(M_p)}({\mathbb {R}})$ are the spaces of ultradifferentiable functions defined by weights and sequences (for the definitions see [11]). We fix a sequence $(M_p)$ and prove that $\mathcal {S}_{(\omega )}({\mathbb {R}})\ne \mathcal {S}_{(M_p)}({\mathbb {R}})$. Clearly, we can assume that $(M_p)$ is non-quasianalytic since the weight $\omega$ is non-quasianalytic. In particular, $(M_p)$ satisfies (*M*0) (see [11], condition $(M3)'$, and use also (*M*1)). If $f\in \mathcal {E}_{(M_p)}({\mathbb {R}})\setminus \mathcal {E}_{(\omega )}({\mathbb {R}})$, then there are a compact set $K\subseteq {\mathbb {R}}$ and $m\in {\mathbb {N}}$ such that

$$\begin{aligned} \sup _{j\in {\mathbb {N}}_0}\sup _{x\in K}| f^{(j)}(x)|e^{-m\varphi ^*_{\omega } \left( \frac{j}{m}\right) }=+\infty . \end{aligned}$$

Hence,

$$\begin{aligned} \forall n\in {\mathbb {N}}\ \exists x_n\in K, j_n\in {\mathbb {N}}\ \text{ such } \text{ that } |f^{(j_n)}(x_n)|\ge ne^{m\varphi ^*_{\omega }\left( \frac{j_n}{m}\right) }. \end{aligned}$$

Since *K* is compact we can assume that the sequence $(x_n)$ converges to some $x_0\in K$. Let $\varphi \in \mathcal {D}_{(M_p)}({\mathbb {R}})$ (the space of functions in $\mathcal {E}_{(M_p)}({\mathbb {R}})$ with compact support) with $\varphi \equiv 1$ in a neighbourhood of $x_0$. Then $g=f\varphi \in \mathcal {D}_{(M_p)}({\mathbb {R}})\subseteq \mathcal {S}_{(M_p)}({\mathbb {R}})$ but, for *n* sufficiently large,

$$\begin{aligned} \frac{|g^{(j_n)}(x_n)|}{e^{m\varphi ^*_{\omega }\left( \frac{j_n}{m}\right) }}= \frac{|f^{(j_n)}(x_n)|}{e^{m\varphi ^*_{\omega }\left( \frac{j_n}{m}\right) }}\ge n\longrightarrow +\infty , \end{aligned}$$

and hence $g\notin \mathcal {S}_{(\omega )}({\mathbb {R}})$ (see the definition of $\mathcal {S}_{(\omega )}({\mathbb {R}})$ above).

Analogously, for $f\in \mathcal {E}_{(\omega )}({\mathbb {R}}) \setminus \mathcal {E}_{(M_p)}({\mathbb {R}})$ we can construct $g\in \mathcal {S}_{(\omega )}({\mathbb {R}}) \setminus \mathcal {S}_{(M_p)}({\mathbb {R}})$.

The same arguments are valid for the Roumieu case and for dimension bigger than one (considering always isotropic classes).

The following Lemma was proved in dimension 1 in [25, Lemma 2.5]; here we give a version of it in dimension *d*.

### Lemma 12

*Let* $\omega$ *be a weight function. Then there exists a constant* $B>0$ *and, for every* $\lambda >0$, *there exists* $C_\lambda >0$, *such that*

6.3 $$\begin{aligned} \lambda \omega _{\mathbf {W}^{(\lambda )}}(t)\le \omega (t)\le B\lambda \omega _{\mathbf {W}^{(\lambda )}}(t)+C_\lambda ,\qquad t\in {\mathbb {R}}^d. \end{aligned}$$

### Proof

For $t=0$ the thesis is trivial, so we can consider $t\ne 0$. Since $|t^\alpha |\le |t|^{|\alpha |}$ for every multi-index $\alpha$, we have

$$\begin{aligned} \lambda \omega _{\mathbf {W}^{(\lambda )}}(t)= & {} \lambda \sup _{\alpha \in {\mathbb {N}}^d_{0,t}} \log \frac{|t^\alpha |}{e^{\varphi ^*_\omega (\lambda |\alpha |)/\lambda }}\le \sup _{\alpha \in {\mathbb {N}}^d_{0,t}}\left\{ \lambda |\alpha |\log |t| -\varphi ^*_\omega (\lambda |\alpha |)\right\} \\\le & {} \varphi ^{**}_\omega (\log |t|) = \omega (t), \end{aligned}$$

so the first inequality of (6.3) is proved. Now, similar to [37, proof of Lemma 5.7], we can prove that, for every $t\in {\mathbb {R}}^d$ such that $|t|\ge e^{\varphi ^*_\omega (\lambda )/\lambda }$,

6.4 $$\begin{aligned} \omega (t)\le 2\sup _{M\in {\mathbb {N}}_0}\left\{ \lambda M\log |t|-\varphi ^*_\omega (\lambda M)\right\} . \end{aligned}$$

Observe now that for every $t\in {\mathbb {R}}^d$, we have $|t|\le \sqrt{d} |t|_\infty \le d |t|_\infty .$ Then by [8, Remark 2.2(iii)],

6.5 $$\begin{aligned} \omega (t)\le \omega (d|t|_\infty )\le D_d\left( \omega (|t|_\infty )+1\right) , \end{aligned}$$

for $D_d=L+L^2+\ldots +L^{d-1}$, where *L* is the constant of condition $(\alpha )$ in Definition 1.

Fix now $t\in {\mathbb {R}}^d$ with $|t|\ge e^{\varphi ^*_\omega (\lambda )/\lambda }$ and let $j_0$ be such that $|t|_\infty =|t_{j_0}|$; for every $M\in {\mathbb {N}}_0$, we then write $\alpha _M:=Me_{j_0}$. We then have $|t|_\infty ^M=|t^{\alpha _M}|$, and so by (6.4) we obtain

$$\begin{aligned} \omega (|t|_\infty )=&\omega (|t_{j_0}|)\le 2\lambda \sup _{M\in {\mathbb {N}}_0} \log \frac{|t^{\alpha _M}|}{e^{\varphi ^*_\omega (\lambda |\alpha _M|)/\lambda }}\\ \le&2\lambda \sup _{\alpha \in {\mathbb {N}}^d_{0,t}} \log \frac{|t^\alpha |}{e^{\varphi ^*_\omega (\lambda |\alpha |)/\lambda }}=2\lambda \omega _{\mathbf {W}^{(\lambda )}}(t), \end{aligned}$$

since $\alpha _M\in {\mathbb {N}}^d_{0,t}$ due to the fact that $t_{j_0}\ne 0$ (we are in fact considering $t\in {\mathbb {R}}^d$ such that $|t|\ge e^{\varphi ^*_\omega (\lambda )/\lambda }$). By (6.5) we then obtain

$$\begin{aligned} \omega (t)\le 2\lambda D_d\omega _{\mathbf {W}^{(\lambda )}}(t)+D_d \end{aligned}$$

for $|t|\ge e^{\varphi ^*_\omega (\lambda )/\lambda }$. Then the second inequality of (6.3) holds for

$$\begin{aligned} B=2D_d\quad \text {and}\quad C_\lambda =D_d+\sup _{|t|\le e^{\varphi ^*_\omega (\lambda )/\lambda }}\omega (t). \end{aligned}$$

$$\square$$

### Lemma 13

*Let* $\omega$ *be a weight function and consider the weight matrix* $\mathcal {M}_\omega$ *as defined in* (6.1), (6.2). *Then for* $r>0$:

- $\omega (t)=O(t^{1/r})$ *as* $t\rightarrow +\infty$ *if and only if*
  6.6 $$\begin{aligned} \forall \,\lambda >0\ \exists \, C,D\ge 1\ \forall \alpha \in {\mathbb {N}}^d:\ \alpha ^{r\alpha }\le CD^{|\alpha |}W^{(\lambda )}_\alpha ; \end{aligned}$$
- $\omega (t)=o(t^{1/r})$ as $t\rightarrow +\infty$ *if and only if*
  6.7 $$\begin{aligned} \forall \,\lambda ,D>0\ \exists \, C\ge 1\ \forall \alpha \in {\mathbb {N}}^d:\ \alpha ^{r\alpha }\le CD^{|\alpha |}W^{(\lambda )}_\alpha . \end{aligned}$$

*Moreover, in the conditions above we can replace* “$\ \forall \,\lambda$” *by* “$\ \exists \,\lambda$”.

### Proof

We only consider the case “$\ \forall \,\lambda$” since the proof for the case “$\ \exists \,\lambda$” is analogous.

(*a*): If $\omega (t)=O(t^{1/r})$ as $t\rightarrow +\infty$, there exists $c\ge 1$ such that

$$\begin{aligned} \omega (t)\le ct^{1/r}+c,\qquad t\ge 0, \end{aligned}$$

and hence

$$\begin{aligned} \varphi _\omega (y)=\omega (e^y)\le ce^{y/r}+c,\qquad y\ge 0. \end{aligned}$$

Then

6.8 $$\begin{aligned} \varphi ^*_\omega (x)=&\sup _{y\ge 0}\{xy-\varphi _\omega (y)\} \ge \sup _{y\ge 0}\{xy-ce^{y/r}\}-c\nonumber \\ =&xr\left( \log \frac{xr}{c}-1\right) -c,\qquad \text{ if }\ x\ge \frac{c}{r}\,. \end{aligned}$$

Therefore, for every $\lambda >0$ and $j\in {\mathbb {N}}$ with $j\ge \frac{c}{r\lambda }$, choosing $x=\lambda j$ and multiplying by $1/\lambda$ in (6.8), we have

$$\begin{aligned} \frac{1}{\lambda }\varphi ^*_\omega (\lambda j)\ge jr\left( \log \frac{\lambda jr}{c}-1\right) -\frac{c}{\lambda }=\log j^{jr}+jr\log \frac{\lambda r}{ec}-\frac{c}{\lambda }\end{aligned}$$

and hence, for $j\ge \frac{c}{r\lambda }$,

6.9 $$\begin{aligned} j^{jr}\le e^{\frac{1}{\lambda }\varphi ^*_\omega (\lambda j)} \left( \frac{ec}{\lambda r}\right) ^{jr}e^{\frac{c}{\lambda }} \le \tilde{C}_\lambda D_\lambda ^j\tilde{W}^{(\lambda )}_j \end{aligned}$$

for $\tilde{C}_\lambda =e^{c/\lambda }$, $D_\lambda =\max \left\{ \left( \frac{ec}{\lambda r}\right) ^r,1\right\}$, and $\tilde{W}^{(\lambda )}_j=e^{\varphi ^*_\omega (\lambda j)/\lambda }$. Enlarging the constants $\tilde{C}_\lambda ,D_\lambda$ we have (6.9) for all $j\in {\mathbb {N}}$. Then

$$\begin{aligned} \alpha ^{r\alpha }=\alpha _1^{r\alpha _1}\dots \alpha _d^{r\alpha _d}\le \tilde{C}_\lambda D_\lambda ^{\alpha _1}\tilde{W}^{(\lambda )}_{\alpha _1}\dots \tilde{C}_\lambda D_\lambda ^{\alpha _d}\tilde{W}^{(\lambda )}_{\alpha _d}, \end{aligned}$$

and so we obtain (6.6) for $C=\tilde{C}_\lambda ^d$ in view of Lemma 11(vii).

Conversely, if (6.6) holds then, by definition of associated function we obtain, for $z\in {\mathbb {R}}^d$,

$$\begin{aligned} \omega _{\mathbf {W}^{(\lambda )}}(z)=&\sup _{\alpha \in {\mathbb {N}}^d_{0,z}} \log \frac{|z^\alpha |}{W^{(\lambda )}_\alpha } \le \sup _{\alpha \in {\mathbb {N}}^d_{0,z}}\log |z^\alpha |\frac{CD^{|\alpha |}}{\alpha ^{r\alpha }}\\ \le&\sup _{\alpha \in {\mathbb {N}}^d_{0,z}}\left( \log C+\sum _{j=1}^d \log \frac{(|z_j| D)^{\alpha _j}}{\alpha _j^{r\alpha _j}}\right) . \end{aligned}$$

Consider now *j* such that $z_j\ne 0$ (otherwise the corresponding addend in the previous sum is 0). A simple computation shows that

$$\begin{aligned} \sup _{\alpha _j\in {\mathbb {N}}}\log \frac{(|z_j| D)^{\alpha _j}}{\alpha _j^{r\alpha _j}}\le \sup _{s>0}\log \frac{(|z_j| D)^{s}}{s^{rs}}\le \frac{r}{e}(|z_j| D)^{1/r}. \end{aligned}$$

We then have

6.10 $$\begin{aligned} \omega _{\mathbf {W}^{(\lambda )}}(z)\le \log C+\sum _{j=1}^d \frac{r}{e}(|z_j| D)^{1/r}\le \log C+\frac{dr}{e}(|z| D)^{1/r}. \end{aligned}$$

By Lemma 12, we have $\omega (z)=\omega (|z|)=O(|z|^{1/r})$ as $|z|\rightarrow +\infty$ for $z\in {\mathbb {R}}^d$, which is equivalent to $\omega (t)=O(t^{1/r})$ as $t\rightarrow +\infty$ for $t\in {\mathbb {R}}$.

(b): If $\omega (t)=o(t^{1/r})$ as $t\rightarrow +\infty$, then for every $D>0$ there exists $c>0$ such that

$$\begin{aligned} \omega (t)\le Dt^{1/r}+c,\quad t\ge 0. \end{aligned}$$

Proceeding as in (a) we have

$$\begin{aligned} \varphi ^*_\omega (x)\ge xr\left( \log \frac{xr}{D}-1\right) -c, \qquad \text{ for }\ x\ge \frac{D}{r}, \end{aligned}$$

and hence

$$\begin{aligned} \alpha ^{r\alpha }\le e^{c/\lambda }\left( \frac{eD}{\lambda r}\right) ^{r|\alpha |}W^{(\lambda )}_\alpha \end{aligned}$$

and (6.7) is satisfied by the arbitrariness of $D>0$.

Conversely, if (6.7) holds then, proceeding as in (*a*), we have that for every $\lambda ,D>0$ there exists $C>0$ such that (6.10) is valid and, therefore, by Lemma 12, $\omega (z)=o(|z|^{1/r})$ as $|z|\rightarrow +\infty$ for $z\in {\mathbb {R}}^d$, or, equivalently, $\omega (t)=o(t^{1/r})$ as $t\rightarrow +\infty$. $\square$

### Corollary 3

*Let* $\omega$ *be a weight function. We have*

- *The Hermite functions belong to* $\mathcal {S}_{\{\omega \}}({\mathbb {R}}^d)$ *if and only if* $\omega (t)=O(t^2)$ *as* $t\rightarrow +\infty$.
- *The Hermite functions belong to* $\mathcal {S}_{(\omega )}({\mathbb {R}}^d)$ *if and only if* $\omega (t)=o(t^2)$ *as* $t\rightarrow +\infty$.

### Proof

By Lemmas 13 and 11 and Proposition 3, $\omega (t)=O(t^2)$ as $t\rightarrow +\infty$ if and only if $\mathcal {M}_\omega$ satisfies (3.2) if and only if the space $\mathcal {S}_{\{\mathcal {M}_\omega \}}({\mathbb {R}}^d)$ contains the Hermite functions; while $\omega (t)=o(t^2)$ as $t\rightarrow +\infty$ if and only if $\mathcal {M}_\omega$ satisfies (3.3) if and only if $\mathcal {S}_{(\mathcal {M}_\omega )}({\mathbb {R}}^d)$ contains the Hermite functions. $\square$

At this point, some considerations are worthy to be expressed. Among all classes of ultradifferentiable functions defined by global estimates, and in particular classes of rapidly decreasing ultradifferentiable functions, the Gel’fand–Shilov spaces $\mathcal {S}_s({\mathbb {R}}^d)$ (Roumieu) and $\Sigma _s({\mathbb {R}}^d)$ (Beurling) have been largely investigated. If $s\ge 1/2$, the space $\mathcal {S}_s({\mathbb {R}}^d)$ consists of those smooth functions *f* such that there is $C>0$ for which for any $\alpha ,\beta \in {\mathbb {N}}_0^d$ and any $x\in {\mathbb {R}}^d$ we have $|x^\alpha \partial ^\beta f(x)|\le C^{|\alpha +\beta |+1}|\alpha +\beta |!^{s}$. While $\Sigma _s({\mathbb {R}}^d)$ is the space of all the smooth functions *f* such that for each $C>0$ there is $D>0$ such that for any $\alpha ,\beta \in {\mathbb {N}}_0^d$ and any $x\in {\mathbb {R}}^d$ we have $|x^\alpha \partial ^\beta f(x)|\le D\,C^{|\alpha +\beta |}|\alpha +\beta |!^{s}$. For $s>1$, they correspond to the Schwartz class in the context of Gevery classes (Roumieu and Beurling). In this setting the value $s=1/2$ is critical since $\mathcal {S}_s({\mathbb {R}}^d)\ne \{0\}$ if and only if $s\ge 1/2$, while $\Sigma _s({\mathbb {R}}^d)\ne \{0\}$ if and only if $s>1/2$ (see [35]). Under the above conditions the Hermite functions constitute a basis for the Gel’fand–Shilov spaces $\mathcal {S}_s({\mathbb {R}}^d)$ and $\Sigma _s({\mathbb {R}}^d)$. In fact, $\mathcal {S}_s({\mathbb {R}}^d)$ and $\Sigma _s({\mathbb {R}}^d)$ are the subspaces of $\mathcal {S}({\mathbb {R}}^d)$ consisting of those functions *f* that can be expressed through Hermite expansions with coefficients $c_\alpha$ satisfying

$$\begin{aligned} |c_\alpha (f)|\le ce^{-r|\alpha |^{1/2s}} \end{aligned}$$

for some $c,r>0$ (for every $r>0$), as was shown by Zhang [41] (see also [13, 29, 36]). The critical exponent $s=1/2$ for the Gel’fand–Shilov spaces is closely related to condition $\omega (t)=O(t^2)$ as $t\rightarrow \infty$ for the space $\mathcal {S}_{\{\omega \}}({\mathbb {R}}^d)$, as we can see in the following corollary, which is a consequence of Lemma 13 applied to $r=1/2$. On the other hand, we observe that the inclusion $\Sigma _{1/2}({\mathbb {R}}^d)\subseteq \mathcal {S}_{(\omega )}({\mathbb {R}}^d)$ trivially holds since $\Sigma _{1/2}({\mathbb {R}}^d)=\{0\}$.

### Corollary 4

*Let* $\omega$ *be a weight function. If* $\omega (t)=O(t^2)$ *as* $t\rightarrow \infty$, *then the Gel’fand–Shilov space* $\mathcal {S}_{1/2}({\mathbb {R}}^d)$ *is continuously embedded in* $\mathcal {S}_{\{\omega \}}({\mathbb {R}}^d)$.

### Proof

We consider the weight function $\omega _2(t)=\max (0,t^2-1)$ and its corresponding weighted matrix as defined in (6.1), i.e. $\mathcal {M}_{\omega _2}=(W^{(\lambda )}_\alpha )_{\lambda >0,\,\alpha \in {\mathbb {N}}_0^d}$, where $W^{(\lambda )}_\alpha :=e^{\frac{1}{\lambda }\varphi ^*_{\omega _2}(\lambda |\alpha |)}$. A straightforward calculation and Stirling’s formula show that there are two constants $A,C>0$ such that for each $\lambda >0$, there are $B_\lambda ,D_\lambda >0$ satisfying that for any $\alpha \in {\mathbb {N}}_0^d$, we have

$$\begin{aligned} B_\lambda A^{|\alpha |} \lambda ^{|\alpha |/2} |\alpha |!^{1/2}\le W^{(\lambda )}_\alpha \le D_\lambda C^{|\alpha |} \lambda ^{|\alpha |/2} |\alpha |!^{1/2}. \end{aligned}$$

This gives immediately that $\mathcal {S}_{1/2}({\mathbb {R}}^d)=\mathcal {S}_{\{\mathcal {M}_{\omega _2}\}}$. Now, by an application of Proposition 5 we get $\mathcal {S}_{1/2}({\mathbb {R}}^d)=\mathcal {S}_{\{\omega _2\}}({\mathbb {R}}^d)$. On the other hand, by Lemma 13 applied in the particular case $r=1/2$ (and again Proposition 5 and Stirling’s formula) we have

$$\begin{aligned} \mathcal {S}_{1/2}({\mathbb {R}}^d)\subseteq \mathcal {S}_{\{\mathcal {M}_{\omega }\}}=\mathcal {S}_{\{\omega \}}({\mathbb {R}}^d), \end{aligned}$$

for every weight function $\omega$ such that $\omega (t)=O(t^2)$ as $t\rightarrow \infty$, which concludes the proof.$\square$

For a weight function $\omega$ we now consider the sequence spaces

$$\begin{aligned}&\Lambda _{\{\omega \}}:=\{\mathbf {c}=(c_\alpha )\in {\mathbb {C}}^{{\mathbb {N}}_0^d}:\ \exists \, j\in {\mathbb {N}},\ \ \Vert \mathbf {c}\Vert _{\omega ,j}:=\sup _{\alpha \in {\mathbb {N}}^d_0}|c_\alpha | e^{\frac{1}{j}\omega (\alpha ^{1/2}/ j)}<+\infty \},\\&\Lambda _{(\omega )}:=\{\mathbf {c}=(c_\alpha )\in {\mathbb {C}}^{{\mathbb {N}}_0^d}:\ \forall \,j\in {\mathbb {N}},\ \ \Vert \mathbf {c}\Vert _{\omega ,1/j}=\sup _{\alpha \in {\mathbb {N}}^d_0}|c_\alpha |e^{j\omega (\alpha ^{1/2} j)}<+\infty \}. \end{aligned}$$

### Proposition 6

*Let* $\omega$ *be a weight function and* $\mathcal {M}_\omega$ *the weight matrix defined by* (6.1), (6.2). *Then* $\Lambda _{\{\omega \}}=\Lambda _{\{\mathcal {M}_\omega \}}$ *and* $\Lambda _{(\omega )}=\Lambda _{(\mathcal {M}_\omega )}$ *and the equalities are also topological*.

### Proof

From Lemma 12 with $\lambda =j$ (and taking $B\in {\mathbb {N}}$), we have

$$\begin{aligned} e^{\frac{1}{Bj}\omega \left( \frac{\alpha ^{1/2}}{Bj}\right) } \le e^{\omega _{\mathbf {W}^{(j)}}\left( \frac{\alpha ^{1/2}}{Bj}\right) +\frac{C_j}{Bj}} \le e^{\frac{C_j}{Bj}}e^{\omega _{\mathbf {W}^{(j)}}({\alpha ^{1/2}}/{j})} \end{aligned}$$

and, conversely, $e^{\omega _{\mathbf {W}^{(j)}}({\alpha ^{1/2}}/{j})}\le e^{\frac{1}{j}\omega (\alpha ^{1/2}/j)}.$ This proves the Roumieu case. Taking $\lambda =1/j$ we prove analogously the Beurling case.$\square$

We now easily deduce the following consequence of Theorem 1.

### Corollary 5

*Let* $\omega$ *be a weight function. The Hermite functions are an absolute Schauder basis in* $\mathcal {S}_{\{\omega \}}({\mathbb {R}}^d)$ *and*

$$\begin{aligned} T:\ \mathcal {S}_{\{\omega \}}({\mathbb {R}}^d)&\longrightarrow \Lambda _{\{\omega \}}\\ f&\longmapsto (\xi _\gamma (f))_{\gamma \in {\mathbb {N}}_0} \end{aligned}$$

*defines an isomorphism.*

*If moreover* $\omega (t)=o(t^2)$ *as* $t\rightarrow +\infty$, *then the Hermite functions are an absolute Schauder basis in* $\mathcal {S}_{(\omega )}({\mathbb {R}}^d)$ *and*

$$\begin{aligned} T:\ \mathcal {S}_{(\omega )}({\mathbb {R}}^d)\longrightarrow \Lambda _{(\omega )} \end{aligned}$$

*as defined above is also an isomorphism*.

We finally have

### Theorem 6

*If* $\omega$ *is a weight function, then* $\mathcal {S}_{\{\omega \}}({\mathbb {R}}^d)$ *is nuclear. If, moreover,* $\omega (t)=o(t^2)$ *as* $t\rightarrow +\infty$, *then* $\mathcal {S}_{(\omega )}({\mathbb {R}}^d)$ *is nuclear*.
